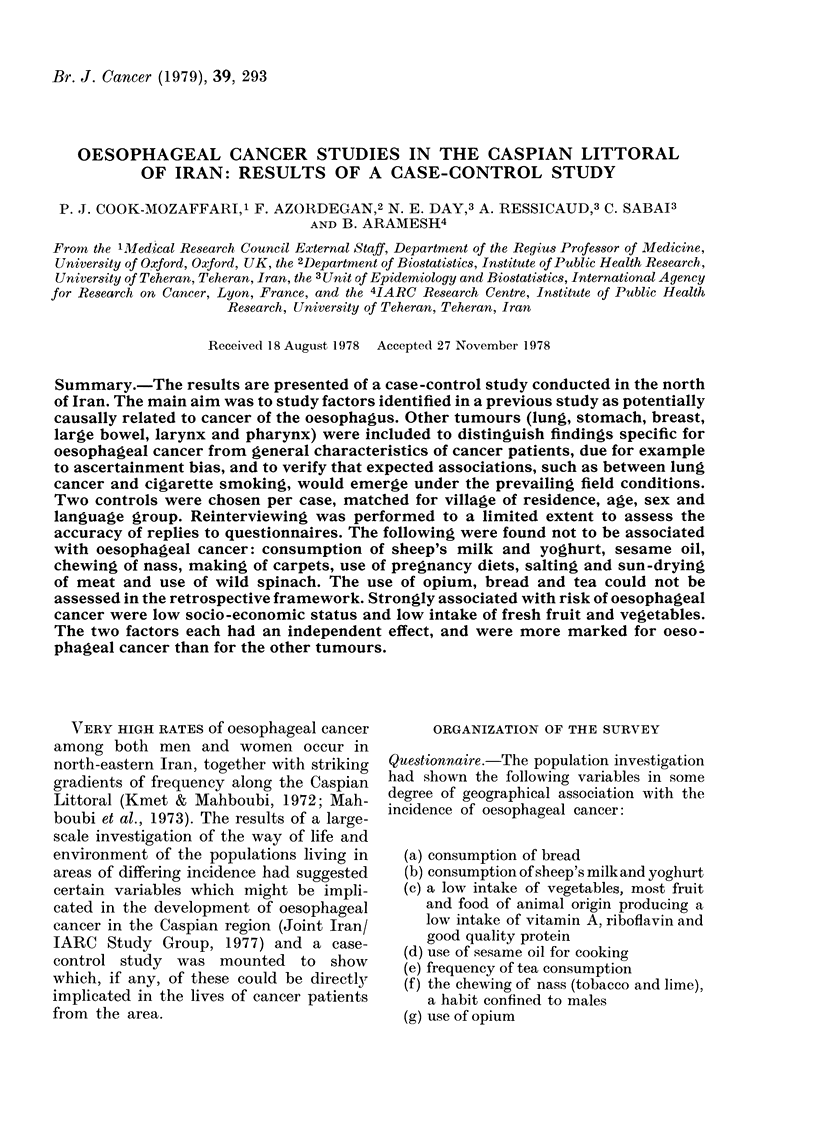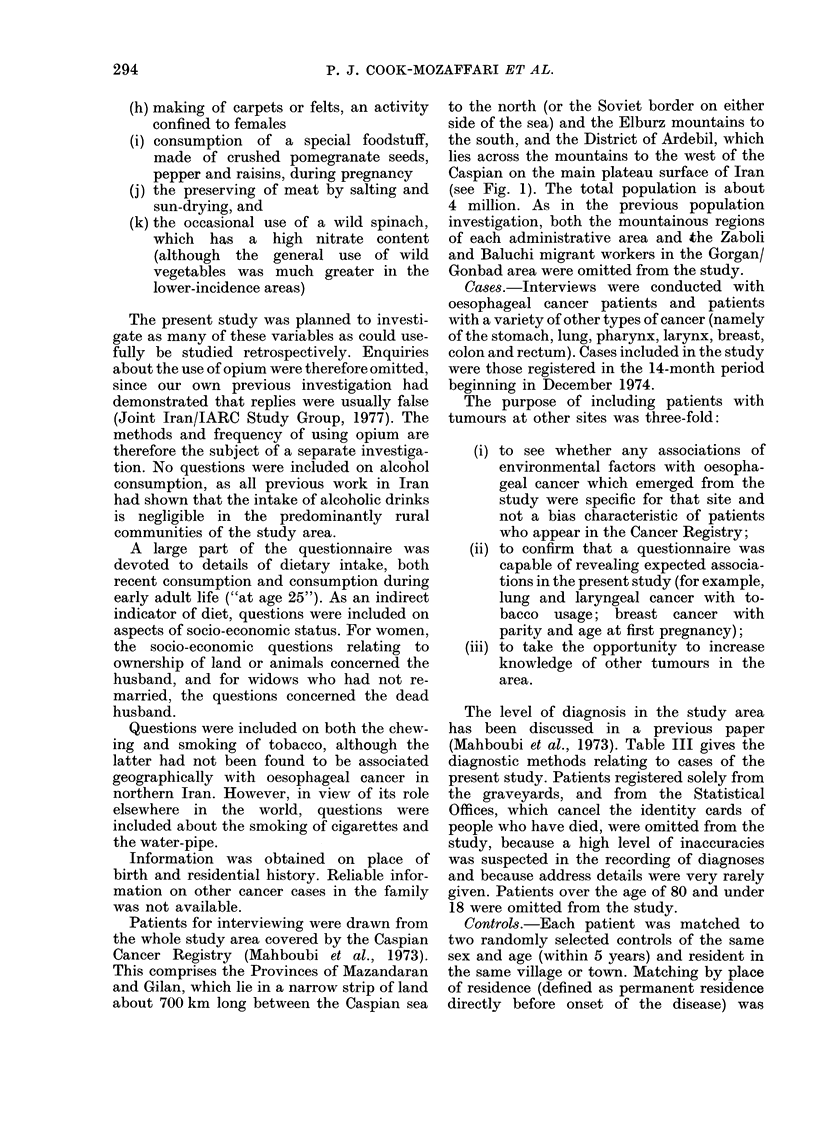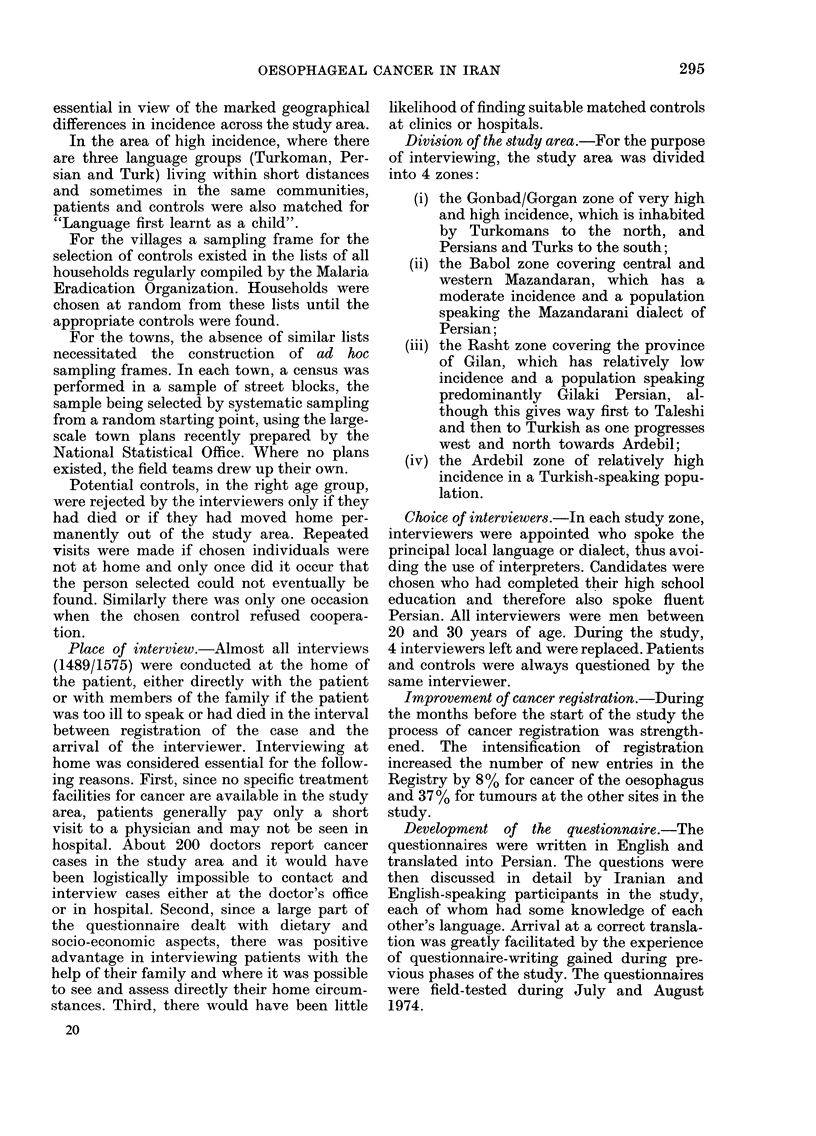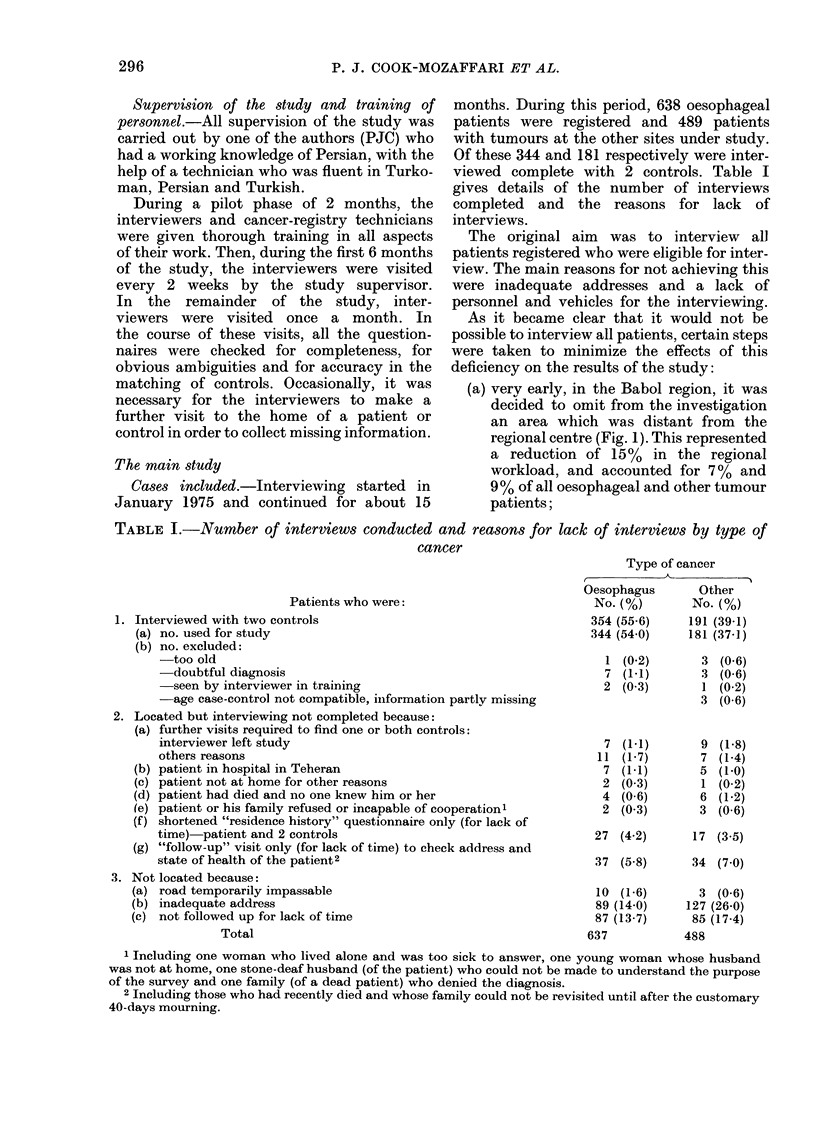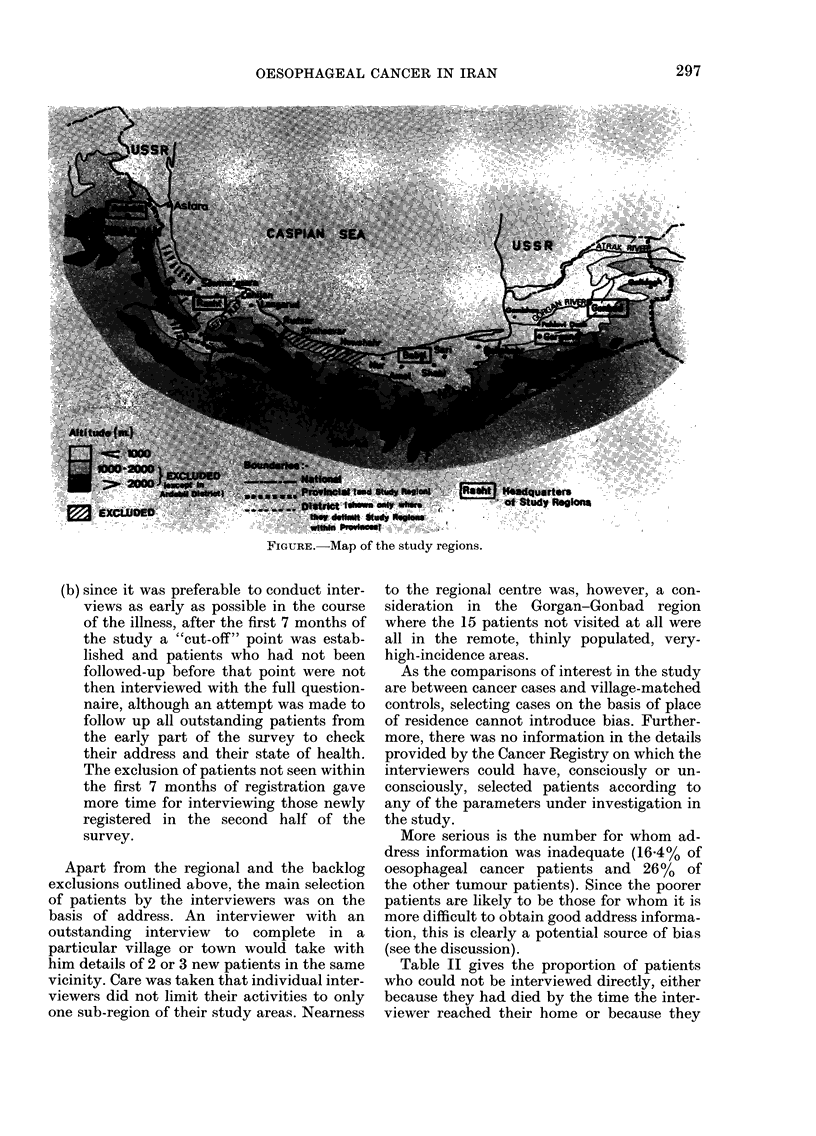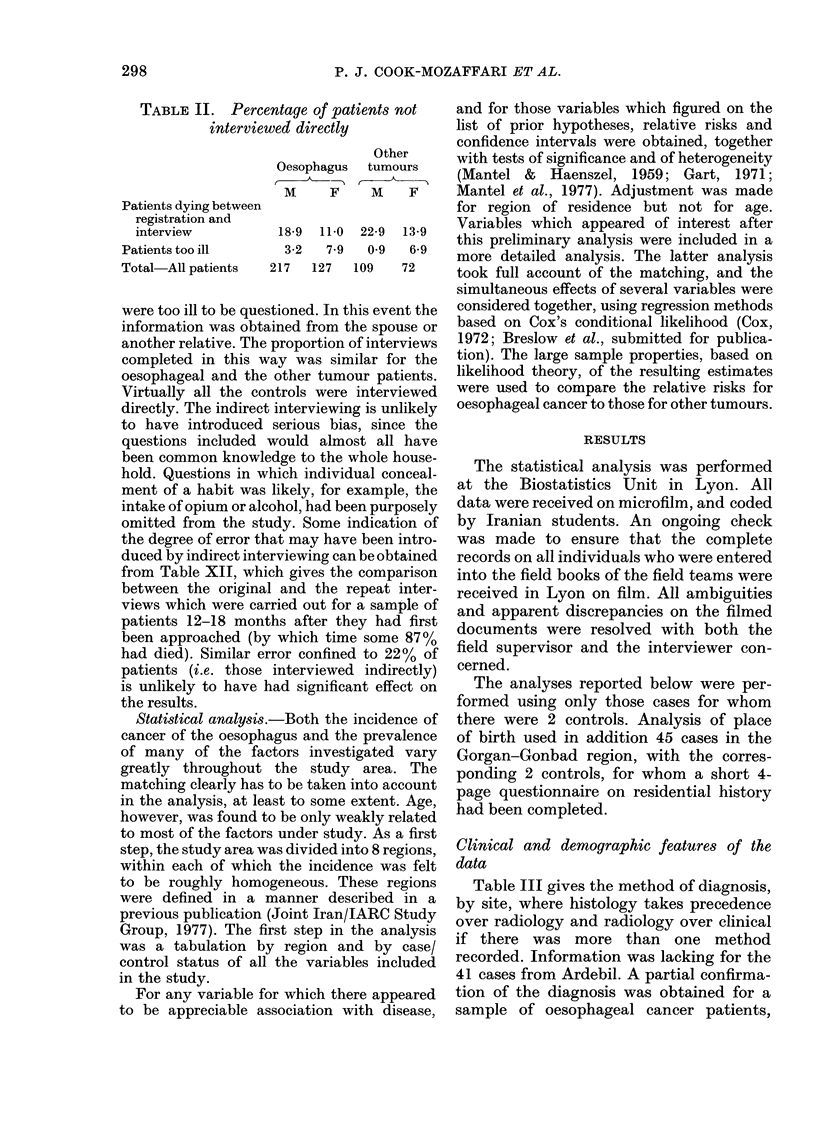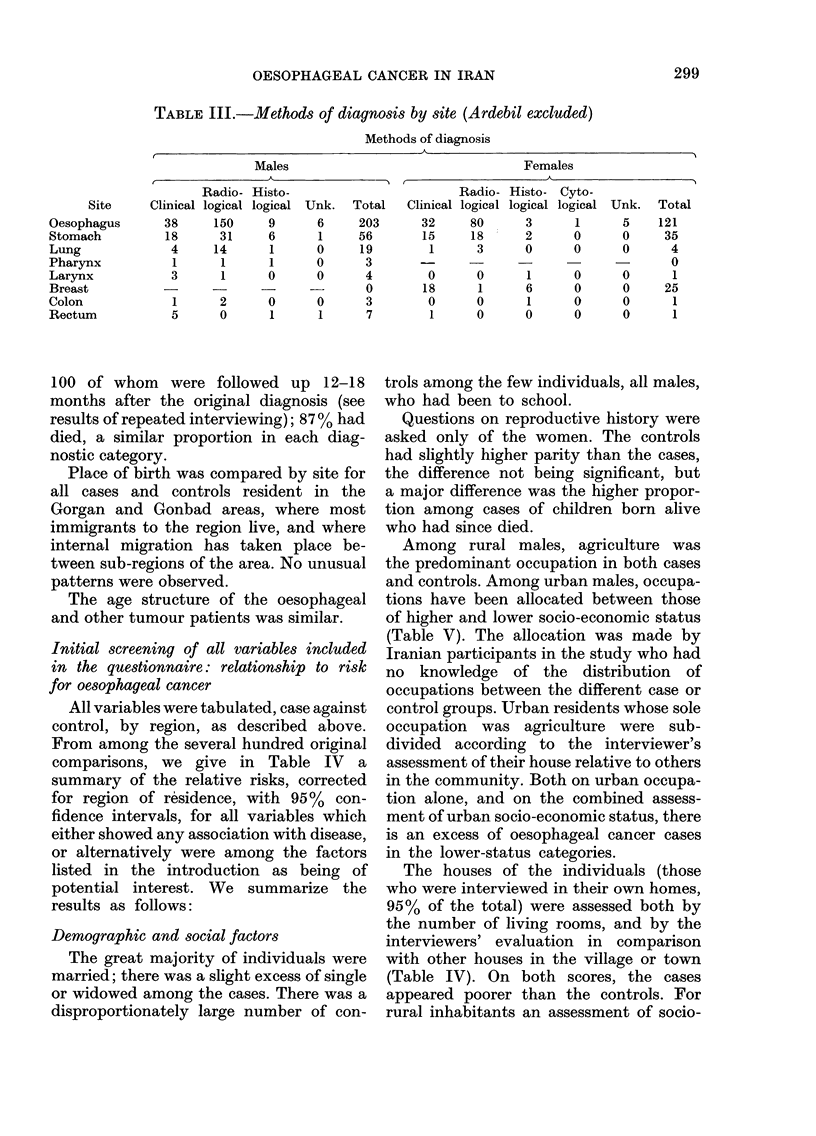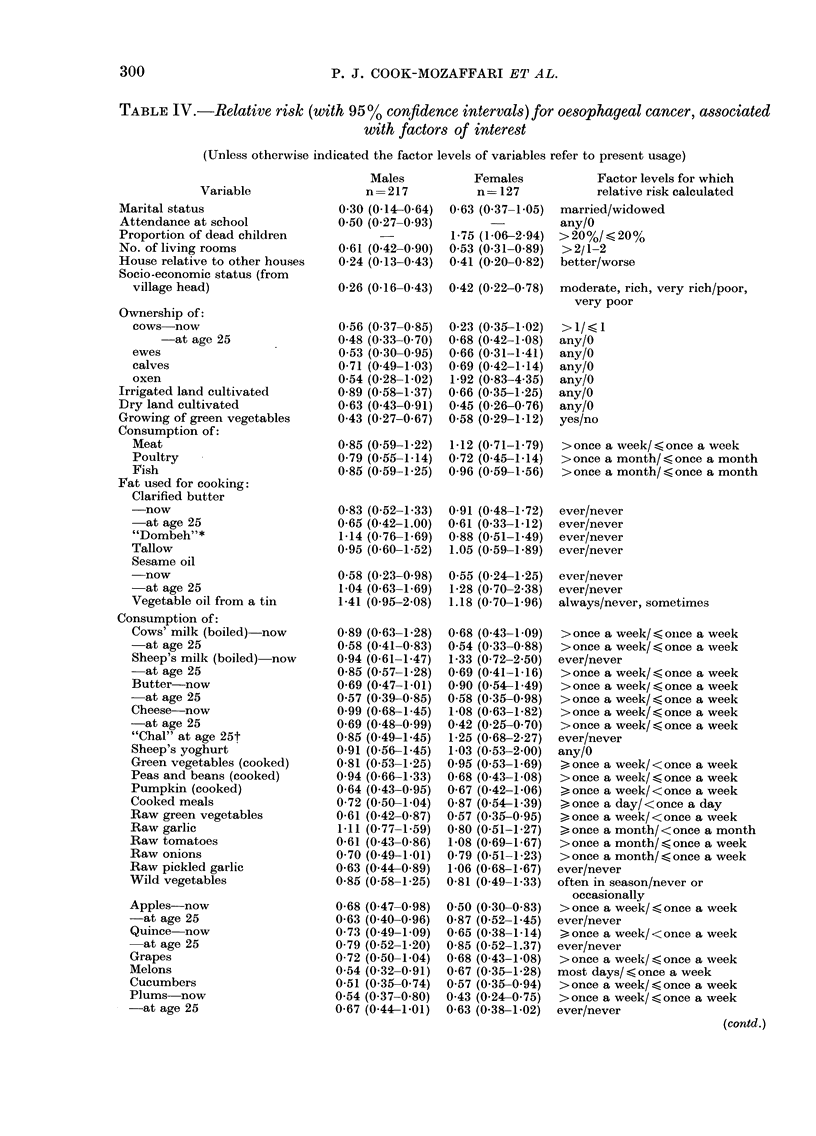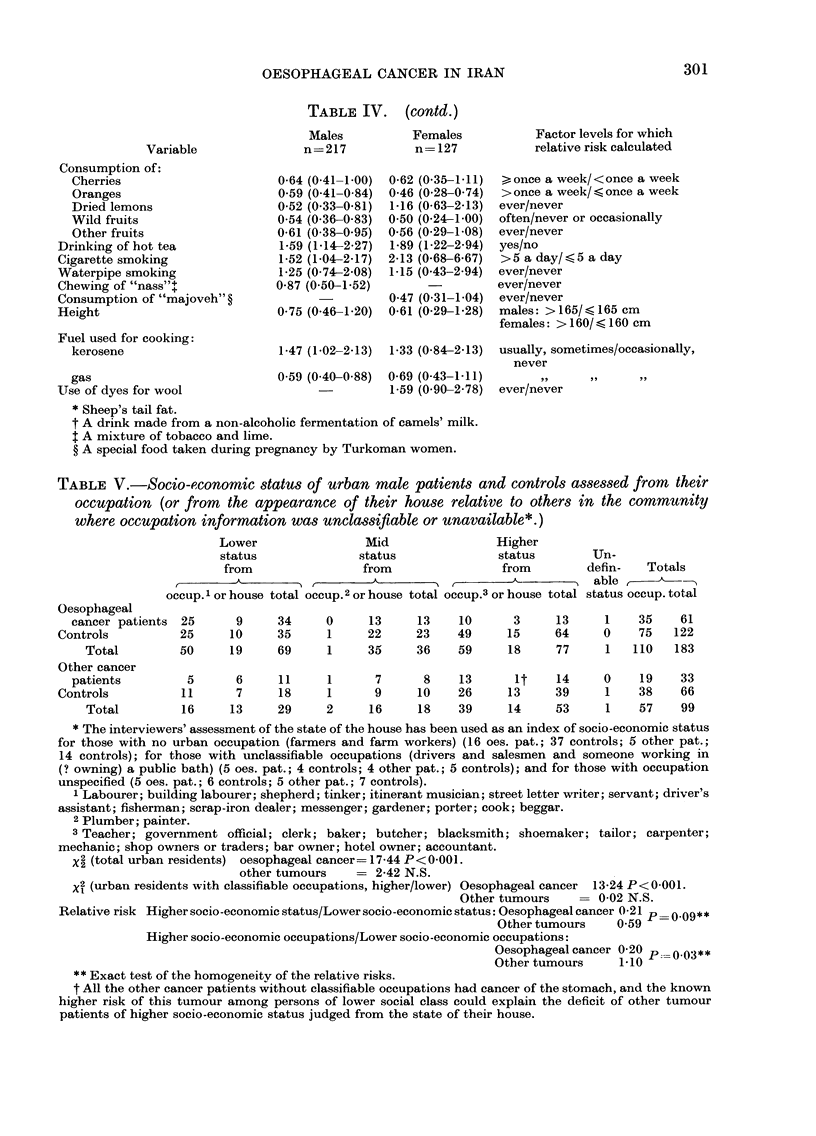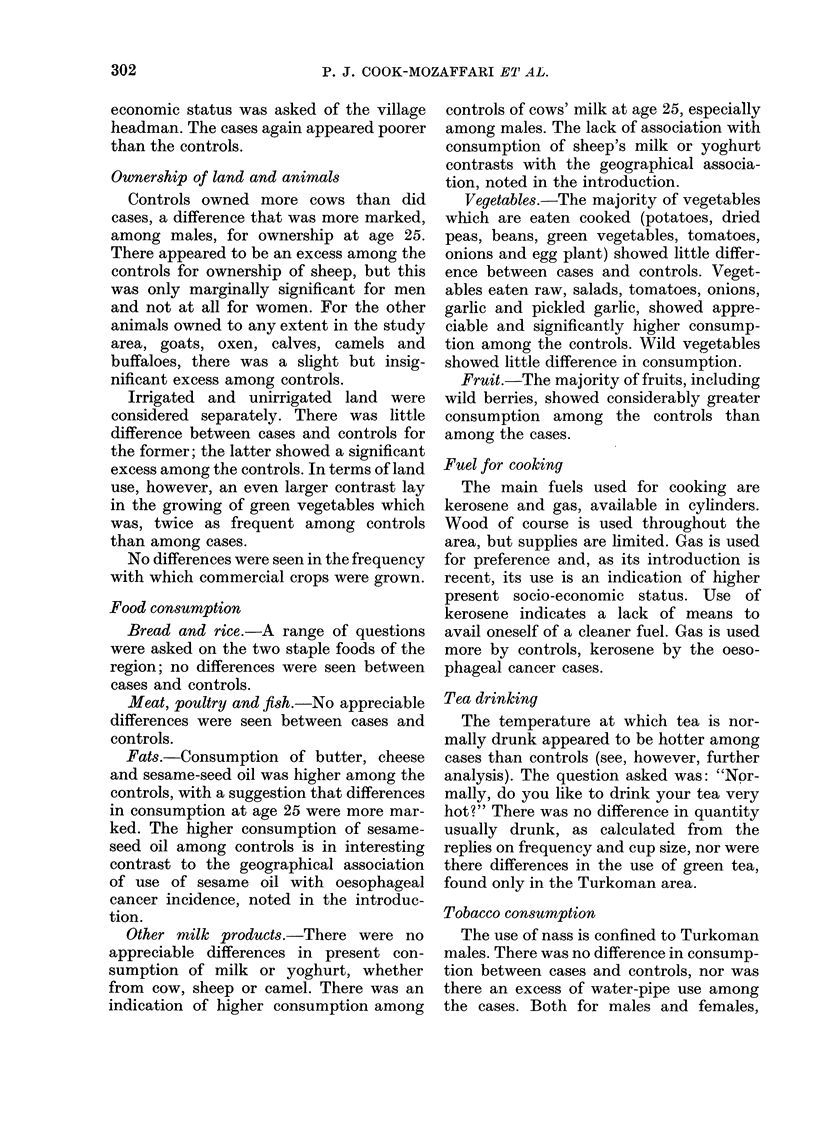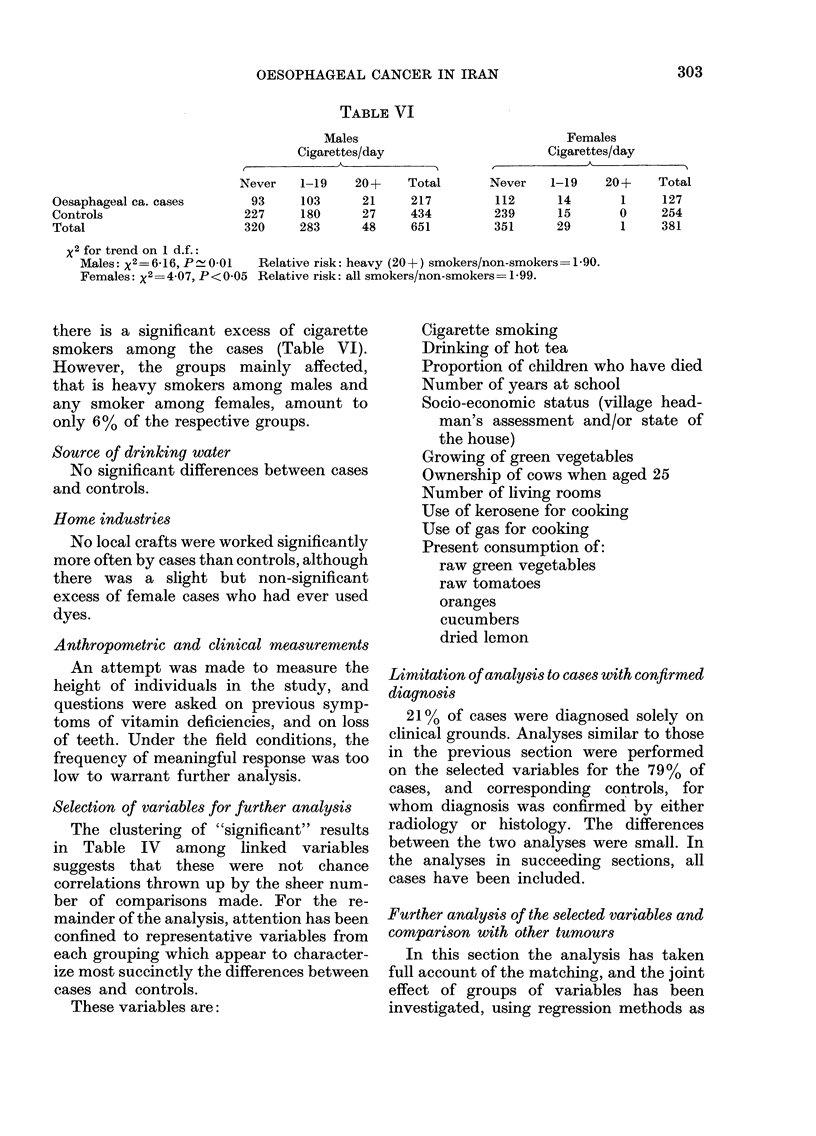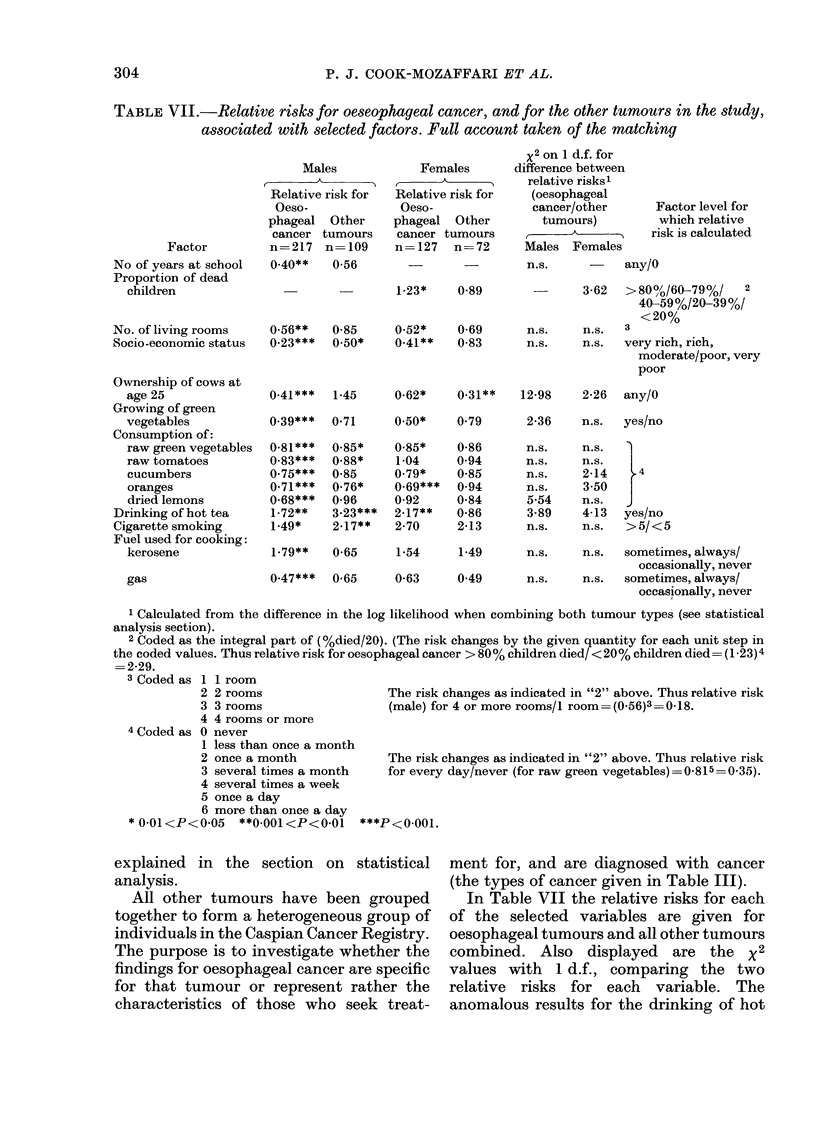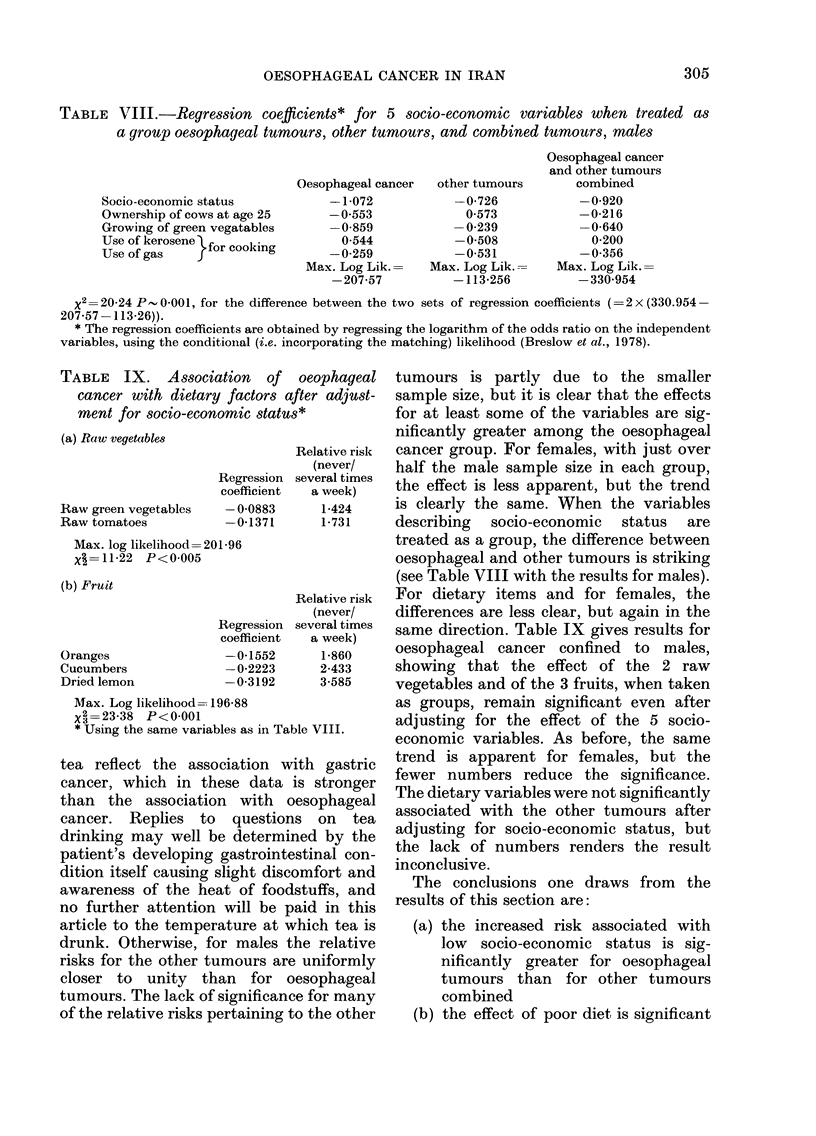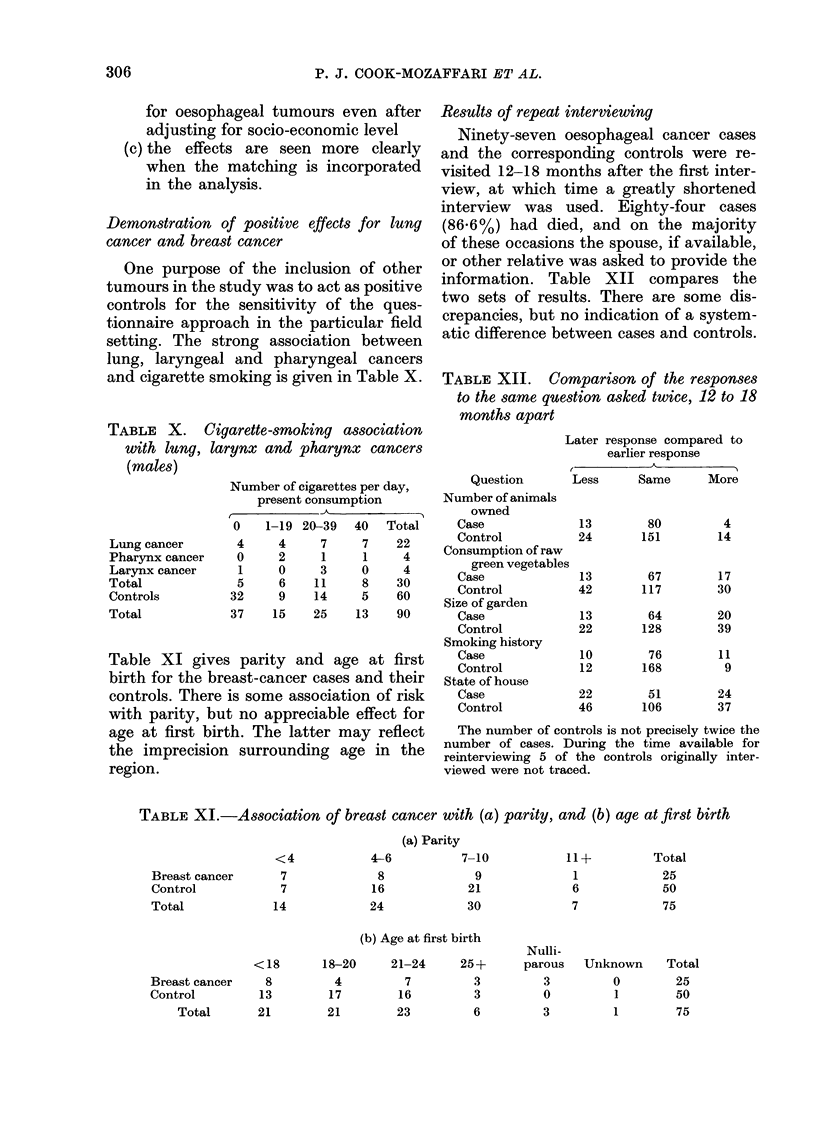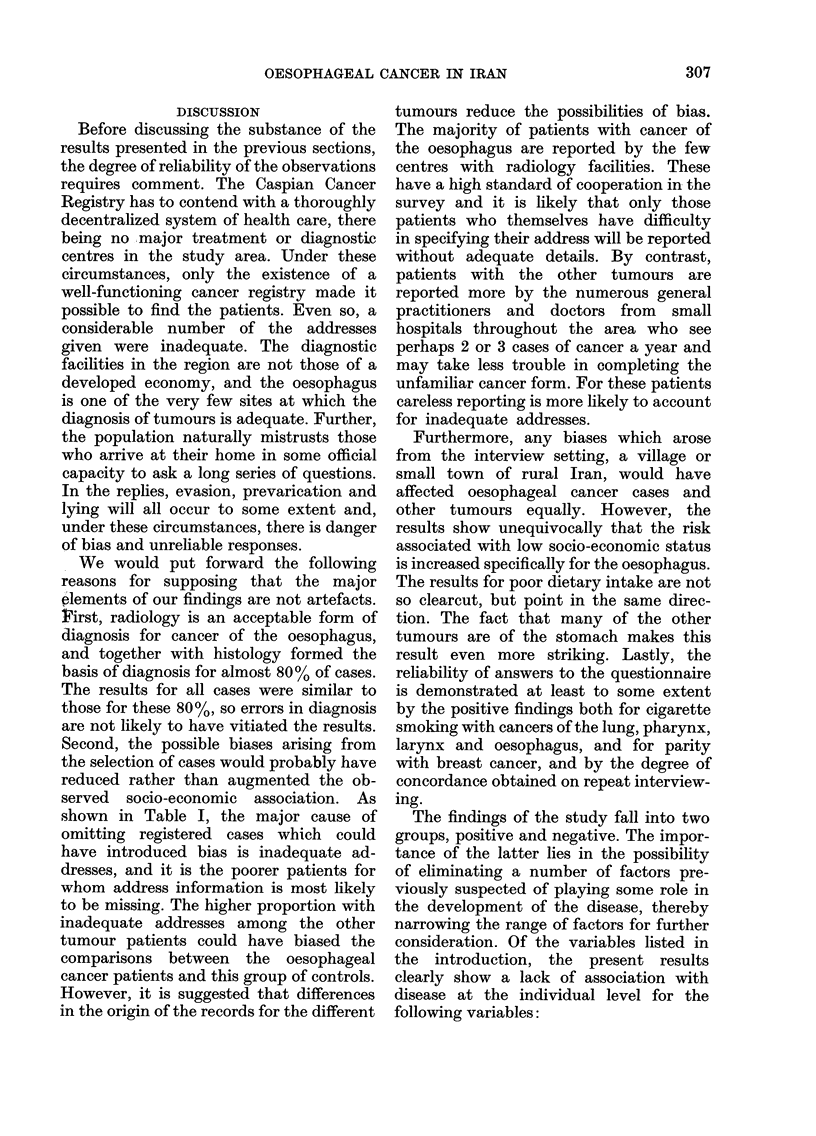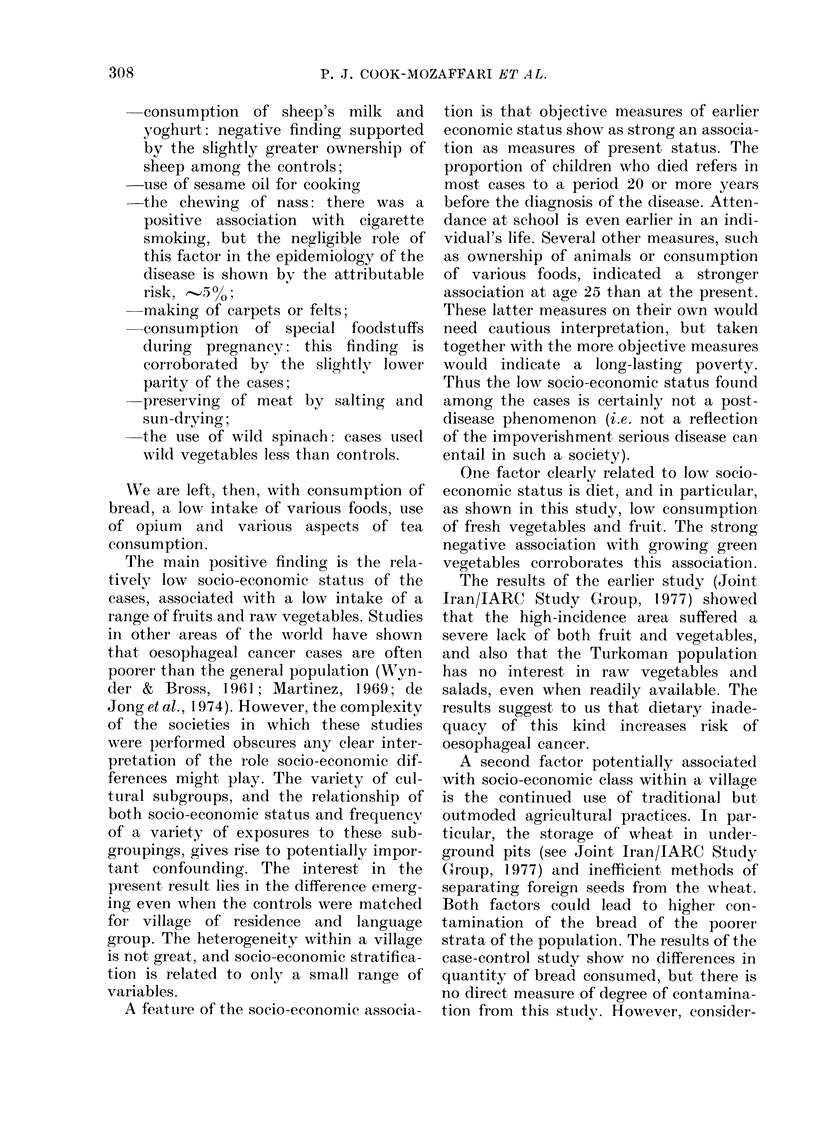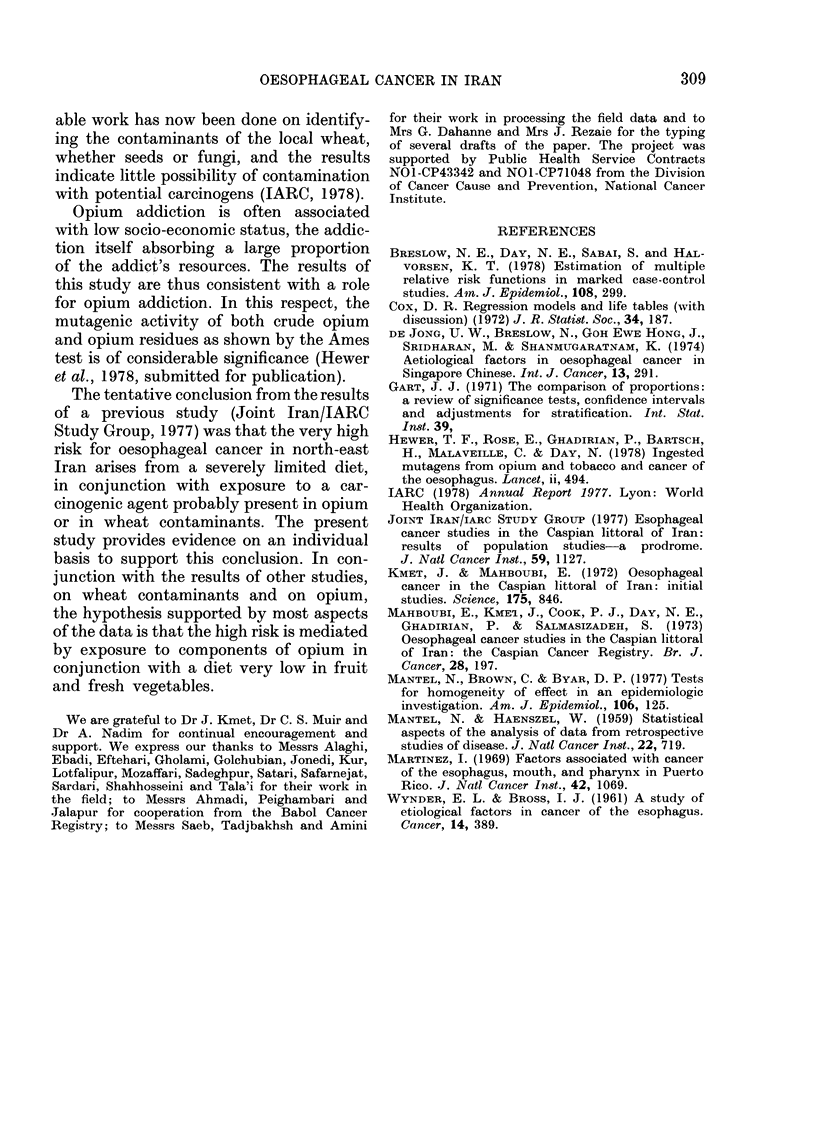# Oesophageal cancer studies in the Caspian Littoral of Iran: results of a case-control study.

**DOI:** 10.1038/bjc.1979.54

**Published:** 1979-03

**Authors:** P. J. Cook-Mozaffari, F. Azordegan, N. E. Day, A. Ressicaud, C. Sabai, B. Aramesh

## Abstract

**Images:**


*Br. J. Cancer* (1979), **39**, 293

### OESOPHAGEAL CANCER STUDIES IN THE CASPIAN LITTORAL OF IRAN: RESULTS OF A CASE-CONTROL STUDY

P. J. COOK-MOZAFFARI,[1] F. AZORDEGAN,[2] N. E. DAY,[3] A. RESSICAUD,[3] C. SABAI[3] AND B. ARAMESH[4]

*From the [1]Medical Research Council External Staff, Department of the Regius Professor of Medicine, University of Oxford, Oxford, UK, the [2]Department of Biostatistics, Institute of Public Health Research, University of Teheran, Teheran, Iran, the [3]Unit of Epidemiology and Biostatistics, International Agency for Research on Cancer, Lyon, France, and the [4]IARC Research Centre, Institute of Public Health Research, University of Teheran, Teheran, Iran*

Received 18 August 1978 Accepted 27 November 1978

**Summary.—The results are presented of a case-control study conducted in the north of Iran. The main aim was to study factors identified in a previous study as potentially causally related to cancer of the oesophagus. Other tumours (lung, stomach, breast, large bowel, larynx and pharynx) were included to distinguish findings specific for oesophageal cancer from general characteristics of cancer patients, due for example to ascertainment bias, and to verify that expected associations, such as between lung cancer and cigarette smoking, would emerge under the prevailing field conditions. Two controls were chosen per case, matched for village of residence, age, sex and language group. Reinterviewing was performed to a limited extent to assess the accuracy of replies to questionnaires. The following were found not to be associated with oesophageal cancer: consumption of sheep's milk and yoghurt, sesame oil, chewing of nass, making of carpets, use of pregnancy diets, salting and sun-drying of meat and use of wild spinach. The use of opium, bread and tea could not be assessed in the retrospective framework. Strongly associated with risk of oesophageal cancer were low socio-economic status and low intake of fresh fruit and vegetables. The two factors each had an independent effect, and were more marked for oesophageal cancer than for the other tumours.**

VERY HIGH RATES of oesophageal cancer among both men and women occur in north-eastern Iran, together with striking gradients of frequency along the Caspian Littoral (Kmet & Mahboubi, 1972; Mahboubi *et al.*, 1973). The results of a large-scale investigation of the way of life and environment of the populations living in areas of differing incidence had suggested certain variables which might be implicated in the development of oesophageal cancer in the Caspian region (Joint Iran/IARC Study Group, 1977) and a case-control study was mounted to show which, if any, of these could be directly implicated in the lives of cancer patients from the area.

### ORGANIZATION OF THE SURVEY

*Questionnaire.*—The population investigation had shown the following variables in some degree of geographical association with the incidence of oesophageal cancer:

(a) consumption of bread
(b) consumption of sheep's milk and yoghurt
(c) a low intake of vegetables, most fruit and food of animal origin producing a low intake of vitamin A, riboflavin and good quality protein
(d) use of sesame oil for cooking
(e) frequency of tea consumption
(f) the chewing of nass (tobacco and lime), a habit confined to males
(g) use of opium

(h) making of carpets or felts, an activity confined to females
(i) consumption of a special foodstuff, made of crushed pomegranate seeds, pepper and raisins, during pregnancy
(j) the preserving of meat by salting and sun-drying, and
(k) the occasional use of a wild spinach, which has a high nitrate content (although the general use of wild vegetables was much greater in the lower-incidence areas)

The present study was planned to investigate as many of these variables as could usefully be studied retrospectively. Enquiries about the use of opium were therefore omitted, since our own previous investigation had demonstrated that replies were usually false (Joint Iran/IARC Study Group, 1977). The methods and frequency of using opium are therefore the subject of a separate investigation. No questions were included on alcohol consumption, as all previous work in Iran had shown that the intake of alcoholic drinks is negligible in the predominantly rural communities of the study area.

A large part of the questionnaire was devoted to details of dietary intake, both recent consumption and consumption during early adult life ("at age 25"). As an indirect indicator of diet, questions were included on aspects of socio-economic status. For women, the socio-economic questions relating to ownership of land or animals concerned the husband, and for widows who had not remarried, the questions concerned the dead husband.

Questions were included on both the chewing and smoking of tobacco, although the latter had not been found to be associated geographically with oesophageal cancer in northern Iran. However, in view of its role elsewhere in the world, questions were included about the smoking of cigarettes and the water-pipe.

Information was obtained on place of birth and residential history. Reliable information on other cancer cases in the family was not available.

Patients for interviewing were drawn from the whole study area covered by the Caspian Cancer Registry (Mahboubi *et al.*, 1973). This comprises the Provinces of Mazandaran and Gilan, which lie in a narrow strip of land about 700 km long between the Caspian sea to the north (or the Soviet border on either side of the sea) and the Elburz mountains to the south, and the District of Ardebil, which lies across the mountains to the west of the Caspian on the main plateau surface of Iran (see Fig. 1). The total population is about 4 million. As in the previous population investigation, both the mountainous regions of each administrative area and the Zaboli and Baluchi migrant workers in the Gorgan/Gonbad area were omitted from the study.

*Cases*.—Interviews were conducted with oesophageal cancer patients and patients with a variety of other types of cancer (namely of the stomach, lung, pharynx, larynx, breast, colon and rectum). Cases included in the study were those registered in the 14-month period beginning in December 1974.

The purpose of including patients with tumours at other sites was three-fold:

(i) to see whether any associations of environmental factors with oesophageal cancer which emerged from the study were specific for that site and not a bias characteristic of patients who appear in the Cancer Registry;
(ii) to confirm that a questionnaire was capable of revealing expected associations in the present study (for example, lung and laryngeal cancer with tobacco usage; breast cancer with parity and age at first pregnancy);
(iii) to take the opportunity to increase knowledge of other tumours in the area.

The level of diagnosis in the study area has been discussed in a previous paper (Mahboubi *et al.*, 1973). Table III gives the diagnostic methods relating to cases of the present study. Patients registered solely from the graveyards, and from the Statistical Offices, which cancel the identity cards of people who have died, were omitted from the study, because a high level of inaccuracies was suspected in the recording of diagnoses and because address details were very rarely given. Patients over the age of 80 and under 18 were omitted from the study.

*Controls*.—Each patient was matched to two randomly selected controls of the same sex and age (within 5 years) and resident in the same village or town. Matching by place of residence (defined as permanent residence directly before onset of the disease) was essential in view of the marked geographical differences in incidence across the study area.

In the area of high incidence, where there are three language groups (Turkoman, Persian and Turk) living within short distances and sometimes in the same communities, patients and controls were also matched for "Language first learnt as a child".

For the villages a sampling frame for the selection of controls existed in the lists of all households regularly compiled by the Malaria Eradication Organization. Households were chosen at random from these lists until the appropriate controls were found.

For the towns, the absence of similar lists necessitated the construction of *ad hoc* sampling frames. In each town, a census was performed in a sample of street blocks, the sample being selected by systematic sampling from a random starting point, using the large-scale town plans recently prepared by the National Statistical Office. Where no plans existed, the field teams drew up their own.

Potential controls, in the right age group, were rejected by the interviewers only if they had died or if they had moved home permanently out of the study area. Repeated visits were made if chosen individuals were not at home and only once did it occur that the person selected could not eventually be found. Similarly there was only one occasion when the chosen control refused cooperation.

*Place of interview.*—Almost all interviews (1489/1575) were conducted at the home of the patient, either directly with the patient or with members of the family if the patient was too ill to speak or had died in the interval between registration of the case and the arrival of the interviewer. Interviewing at home was considered essential for the following reasons. First, since no specific treatment facilities for cancer are available in the study area, patients generally pay only a short visit to a physician and may not be seen in hospital. About 200 doctors report cancer cases in the study area and it would have been logistically impossible to contact and interview cases either at the doctor's office or in hospital. Second, since a large part of the questionnaire dealt with dietary and socio-economic aspects, there was positive advantage in interviewing patients with the help of their family and where it was possible to see and assess directly their home circumstances. Third, there would have been little likelihood of finding suitable matched controls at clinics or hospitals.

*Division of the study area.*—For the purpose of interviewing, the study area was divided into 4 zones:

(i) the Gonbad/Gorgan zone of very high and high incidence, which is inhabited by Turkomans to the north, and Persians and Turks to the south;
(ii) the Babol zone covering central and western Mazandaran, which has a moderate incidence and a population speaking the Mazandarani dialect of Persian;
(iii) the Rasht zone covering the province of Gilan, which has relatively low incidence and a population speaking predominantly Gilaki Persian, although this gives way first to Taleshi and then to Turkish as one progresses west and north towards Ardebil;
(iv) the Ardebil zone of relatively high incidence in a Turkish-speaking population.

*Choice of interviewers.*—In each study zone, interviewers were appointed who spoke the principal local language or dialect, thus avoiding the use of interpreters. Candidates were chosen who had completed their high school education and therefore also spoke fluent Persian. All interviewers were men between 20 and 30 years of age. During the study, 4 interviewers left and were replaced. Patients and controls were always questioned by the same interviewer.

*Improvement of cancer registration.*—During the months before the start of the study the process of cancer registration was strengthened. The intensification of registration increased the number of new entries in the Registry by 8% for cancer of the oesophagus and 37% for tumours at the other sites in the study.

*Development of the questionnaire.*—The questionnaires were written in English and translated into Persian. The questions were then discussed in detail by Iranian and English-speaking participants in the study, each of whom had some knowledge of each other's language. Arrival at a correct translation was greatly facilitated by the experience of questionnaire-writing gained during previous phases of the study. The questionnaires were field-tested during July and August 1974.

*Supervision of the study and training of personnel.*—All supervision of the study was carried out by one of the authors (PJC) who had a working knowledge of Persian, with the help of a technician who was fluent in Turkoman, Persian and Turkish.

During a pilot phase of 2 months, the interviewers and cancer-registry technicians were given thorough training in all aspects of their work. Then, during the first 6 months of the study, the interviewers were visited every 2 weeks by the study supervisor. In the remainder of the study, interviewers were visited once a month. In the course of these visits, all the questionnaires were checked for completeness, for obvious ambiguities and for accuracy in the matching of controls. Occasionally, it was necessary for the interviewers to make a further visit to the home of a patient or control in order to collect missing information.

*The main study*

*Cases included.*—Interviewing started in January 1975 and continued for about 15 months. During this period, 638 oesophageal patients were registered and 489 patients with tumours at the other sites under study. Of these 344 and 181 respectively were interviewed complete with 2 controls. Table I gives details of the number of interviews completed and the reasons for lack of interviews.

The original aim was to interview all patients registered who were eligible for interview. The main reasons for not achieving this were inadequate addresses and a lack of personnel and vehicles for the interviewing.

As it became clear that it would not be possible to interview all patients, certain steps were taken to minimize the effects of this deficiency on the results of the study:

(a) very early, in the Babol region, it was decided to omit from the investigation an area which was distant from the regional centre (Fig. 1). This represented a reduction of 15% in the regional workload, and accounted for 7% and 9% of all oesophageal and other tumour patients;

TABLE I.—*Number of interviews conducted and reasons for lack of interviews by type of cancer*

| Patients who were: | Type of cancer: Oesophagus No. (%) | Other No. (%) |
|---|---|---|
| 1. Interviewed with two controls | 354 (55·6) | 191 (39·1) |
| (a) no. used for study | 344 (54·0) | 181 (37·1) |
| (b) no. excluded: | | |
| —too old | 1 (0·2) | 3 (0·6) |
| —doubtful diagnosis | 7 (1·1) | 3 (0·6) |
| —seen by interviewer in training | 2 (0·3) | 1 (0·2) |
| —age case-control not compatible, information partly missing | | 3 (0·6) |
| 2. Located but interviewing not completed because: | | |
| (a) further visits required to find one or both controls: | | |
| interviewer left study | 7 (1·1) | 9 (1·8) |
| others reasons | 11 (1·7) | 7 (1·4) |
| (b) patient in hospital in Teheran | 7 (1·1) | 5 (1·0) |
| (c) patient not at home for other reasons | 2 (0·3) | 1 (0·2) |
| (d) patient had died and no one knew him or her | 4 (0·6) | 6 (1·2) |
| (e) patient or his family refused or incapable of cooperation[1] | 2 (0·3) | 3 (0·6) |
| (f) shortened "residence history" questionnaire only (for lack of time)—patient and 2 controls | 27 (4·2) | 17 (3·5) |
| (g) "follow-up" visit only (for lack of time) to check address and state of health of the patient[2] | 37 (5·8) | 34 (7·0) |
| 3. Not located because: | | |
| (a) road temporarily impassable | 10 (1·6) | 3 (0·6) |
| (b) inadequate address | 89 (14·0) | 127 (26·0) |
| (c) not followed up for lack of time | 87 (13·7) | 85 (17·4) |
| Total | 637 | 488 |

[1] Including one woman who lived alone and was too sick to answer, one young woman whose husband was not at home, one stone-deaf husband (of the patient) who could not be made to understand the purpose of the survey and one family (of a dead patient) who denied the diagnosis.

[2] Including those who had recently died and whose family could not be revisited until after the customary 40-days mourning.

FIGURE.—Map of the study regions.

(b) since it was preferable to conduct interviews as early as possible in the course of the illness, after the first 7 months of the study a "cut-off" point was established and patients who had not been followed-up before that point were not then interviewed with the full questionnaire, although an attempt was made to follow up all outstanding patients from the early part of the survey to check their address and their state of health. The exclusion of patients not seen within the first 7 months of registration gave more time for interviewing those newly registered in the second half of the survey.

Apart from the regional and the backlog exclusions outlined above, the main selection of patients by the interviewers was on the basis of address. An interviewer with an outstanding interview to complete in a particular village or town would take with him details of 2 or 3 new patients in the same vicinity. Care was taken that individual interviewers did not limit their activities to only one sub-region of their study areas. Nearness to the regional centre was, however, a consideration in the Gorgan–Gonbad region where the 15 patients not visited at all were all in the remote, thinly populated, very-high-incidence areas.

As the comparisons of interest in the study are between cancer cases and village-matched controls, selecting cases on the basis of place of residence cannot introduce bias. Furthermore, there was no information in the details provided by the Cancer Registry on which the interviewers could have, consciously or unconsciously, selected patients according to any of the parameters under investigation in the study.

More serious is the number for whom address information was inadequate (16·4% of oesophageal cancer patients and 26% of the other tumour patients). Since the poorer patients are likely to be those for whom it is more difficult to obtain good address information, this is clearly a potential source of bias (see the discussion).

Table II gives the proportion of patients who could not be interviewed directly, either because they had died by the time the interviewer reached their home or because they

TABLE II. *Percentage of patients not interviewed directly*

| | Oesophagus | | Other tumours | |
|---|---|---|---|---|
| | M | F | M | F |
| Patients dying between registration and interview | 18·9 | 11·0 | 22·9 | 13·9 |
| Patients too ill | 3·2 | 7·9 | 0·9 | 6·9 |
| Total—All patients | 217 | 127 | 109 | 72 |

were too ill to be questioned. In this event the information was obtained from the spouse or another relative. The proportion of interviews completed in this way was similar for the oesophageal and the other tumour patients. Virtually all the controls were interviewed directly. The indirect interviewing is unlikely to have introduced serious bias, since the questions included would almost all have been common knowledge to the whole household. Questions in which individual concealment of a habit was likely, for example, the intake of opium or alcohol, had been purposely omitted from the study. Some indication of the degree of error that may have been introduced by indirect interviewing can be obtained from Table XII, which gives the comparison between the original and the repeat interviews which were carried out for a sample of patients 12–18 months after they had first been approached (by which time some 87% had died). Similar error confined to 22% of patients (*i.e.* those interviewed indirectly) is unlikely to have had significant effect on the results.

*Statistical analysis.*—Both the incidence of cancer of the oesophagus and the prevalence of many of the factors investigated vary greatly throughout the study area. The matching clearly has to be taken into account in the analysis, at least to some extent. Age, however, was found to be only weakly related to most of the factors under study. As a first step, the study area was divided into 8 regions, within each of which the incidence was felt to be roughly homogeneous. These regions were defined in a manner described in a previous publication (Joint Iran/IARC Study Group, 1977). The first step in the analysis was a tabulation by region and by case/control status of all the variables included in the study.

For any variable for which there appeared to be appreciable association with disease, and for those variables which figured on the list of prior hypotheses, relative risks and confidence intervals were obtained, together with tests of significance and of heterogeneity (Mantel & Haenszel, 1959; Gart, 1971; Mantel *et al.*, 1977). Adjustment was made for region of residence but not for age. Variables which appeared of interest after this preliminary analysis were included in a more detailed analysis. The latter analysis took full account of the matching, and the simultaneous effects of several variables were considered together, using regression methods based on Cox's conditional likelihood (Cox, 1972; Breslow *et al.*, submitted for publication). The large sample properties, based on likelihood theory, of the resulting estimates were used to compare the relative risks for oesophageal cancer to those for other tumours.

### RESULTS

The statistical analysis was performed at the Biostatistics Unit in Lyon. All data were received on microfilm, and coded by Iranian students. An ongoing check was made to ensure that the complete records on all individuals who were entered into the field books of the field teams were received in Lyon on film. All ambiguities and apparent discrepancies on the filmed documents were resolved with both the field supervisor and the interviewer concerned.

The analyses reported below were performed using only those cases for whom there were 2 controls. Analysis of place of birth used in addition 45 cases in the Gorgan–Gonbad region, with the corresponding 2 controls, for whom a short 4-page questionnaire on residential history had been completed.

### *Clinical and demographic features of the data*

Table III gives the method of diagnosis, by site, where histology takes precedence over radiology and radiology over clinical if there was more than one method recorded. Information was lacking for the 41 cases from Ardebil. A partial confirmation of the diagnosis was obtained for a sample of oesophageal cancer patients,

TABLE III.—*Methods of diagnosis by site (Ardebil excluded)*

| | Methods of diagnosis | | | | | | | | | | |
|---|---|---|---|---|---|---|---|---|---|---|---|
| | Males | | | | | Females | | | | | |
| Site | Clinical | Radio-logical | Histo-logical | Unk. | Total | Clinical | Radio-logical | Histo-logical | Cyto-logical | Unk. | Total |
| Oesophagus | 38 | 150 | 9 | 6 | 203 | 32 | 80 | 3 | 1 | 5 | 121 |
| Stomach | 18 | 31 | 6 | 1 | 56 | 15 | 18 | 2 | 0 | 0 | 35 |
| Lung | 4 | 14 | 1 | 0 | 19 | 1 | 3 | 0 | 0 | 0 | 4 |
| Pharynx | 1 | 1 | 1 | 0 | 3 | — | — | — | — | — | 0 |
| Larynx | 3 | 1 | 0 | 0 | 4 | 0 | 0 | 1 | 0 | 0 | 1 |
| Breast | — | — | — | — | 0 | 18 | 1 | 6 | 0 | 0 | 25 |
| Colon | 1 | 2 | 0 | 0 | 3 | 0 | 0 | 1 | 0 | 0 | 1 |
| Rectum | 5 | 0 | 1 | 1 | 7 | 1 | 0 | 0 | 0 | 0 | 1 |

100 of whom were followed up 12–18 months after the original diagnosis (see results of repeated interviewing); 87% had died, a similar proportion in each diagnostic category.

Place of birth was compared by site for all cases and controls resident in the Gorgan and Gonbad areas, where most immigrants to the region live, and where internal migration has taken place between sub-regions of the area. No unusual patterns were observed.

The age structure of the oesophageal and other tumour patients was similar.

*Initial screening of all variables included in the questionnaire: relationship to risk for oesophageal cancer*

All variables were tabulated, case against control, by region, as described above. From among the several hundred original comparisons, we give in Table IV a summary of the relative risks, corrected for region of residence, with 95% confidence intervals, for all variables which either showed any association with disease, or alternatively were among the factors listed in the introduction as being of potential interest. We summarize the results as follows:

*Demographic and social factors*

The great majority of individuals were married; there was a slight excess of single or widowed among the cases. There was a disproportionately large number of controls among the few individuals, all males, who had been to school.

Questions on reproductive history were asked only of the women. The controls had slightly higher parity than the cases, the difference not being significant, but a major difference was the higher proportion among cases of children born alive who had since died.

Among rural males, agriculture was the predominant occupation in both cases and controls. Among urban males, occupations have been allocated between those of higher and lower socio-economic status (Table V). The allocation was made by Iranian participants in the study who had no knowledge of the distribution of occupations between the different case or control groups. Urban residents whose sole occupation was agriculture were subdivided according to the interviewer's assessment of their house relative to others in the community. Both on urban occupation alone, and on the combined assessment of urban socio-economic status, there is an excess of oesophageal cancer cases in the lower-status categories.

The houses of the individuals (those who were interviewed in their own homes, 95% of the total) were assessed both by the number of living rooms, and by the interviewers' evaluation in comparison with other houses in the village or town (Table IV). On both scores, the cases appeared poorer than the controls. For rural inhabitants an assessment of socio-

TABLE IV.—*Relative risk (with 95% confidence intervals) for oesophageal cancer, associated with factors of interest*

(Unless otherwise indicated the factor levels of variables refer to present usage)

| Variable | Males n=217 | Females n=127 | Factor levels for which relative risk calculated |
|---|---|---|---|
| Marital status | 0·30 (0·14–0·64) | 0·63 (0·37–1·05) | married/widowed |
| Attendance at school | 0·50 (0·27–0·93) | — | any/0 |
| Proportion of dead children | — | 1·75 (1·06–2·94) | >20%/⩽20% |
| No. of living rooms | 0·61 (0·42–0·90) | 0·53 (0·31–0·89) | >2/1–2 |
| House relative to other houses | 0·24 (0·13–0·43) | 0·41 (0·20–0·82) | better/worse |
| Socio-economic status (from village head) | 0·26 (0·16–0·43) | 0·42 (0·22–0·78) | moderate, rich, very rich/poor, very poor |
| Ownership of: | | | |
| cows—now | 0·56 (0·37–0·85) | 0·23 (0·35–1·02) | >1/⩽1 |
| —at age 25 | 0·48 (0·33–0·70) | 0·68 (0·42–1·08) | any/0 |
| ewes | 0·53 (0·30–0·95) | 0·66 (0·31–1·41) | any/0 |
| calves | 0·71 (0·49–1·03) | 0·69 (0·42–1·14) | any/0 |
| oxen | 0·54 (0·28–1·02) | 1·92 (0·83–4·35) | any/0 |
| Irrigated land cultivated | 0·89 (0·58–1·37) | 0·66 (0·35–1·25) | any/0 |
| Dry land cultivated | 0·63 (0·43–0·91) | 0·45 (0·26–0·76) | any/0 |
| Growing of green vegetables | 0·43 (0·27–0·67) | 0·58 (0·29–1·12) | yes/no |
| Consumption of: | | | |
| Meat | 0·85 (0·59–1·22) | 1·12 (0·71–1·79) | >once a week/⩽once a week |
| Poultry | 0·79 (0·55–1·14) | 0·72 (0·45–1·14) | >once a month/⩽once a month |
| Fish | 0·85 (0·59–1·25) | 0·96 (0·59–1·56) | >once a month/⩽once a month |
| Fat used for cooking: | | | |
| Clarified butter | | | |
| —now | 0·83 (0·52–1·33) | 0·91 (0·48–1·72) | ever/never |
| —at age 25 | 0·65 (0·42–1.00) | 0·61 (0·33–1·12) | ever/never |
| "Dombeh"* | 1·14 (0·76–1·69) | 0·88 (0·51–1·49) | ever/never |
| Tallow | 0·95 (0·60–1·52) | 1.05 (0·59–1·89) | ever/never |
| Sesame oil | | | |
| —now | 0·58 (0·23–0·98) | 0·55 (0·24–1·25) | ever/never |
| —at age 25 | 1·04 (0·63–1·69) | 1·28 (0·70–2·38) | ever/never |
| Vegetable oil from a tin | 1·41 (0·95–2·08) | 1.18 (0·70–1·96) | always/never, sometimes |
| Consumption of: | | | |
| Cows' milk (boiled)—now | 0·89 (0·63–1·28) | 0·68 (0·43–1·09) | >once a week/⩽once a week |
| —at age 25 | 0·58 (0·41–0·83) | 0·54 (0·33–0·88) | >once a week/⩽once a week |
| Sheep's milk (boiled)—now | 0·94 (0·61–1·47) | 1·33 (0·72–2·50) | ever/never |
| —at age 25 | 0·85 (0·57–1·28) | 0·69 (0·41–1·16) | >once a week/⩽once a week |
| Butter—now | 0·69 (0·47–1·01) | 0·90 (0·54–1·49) | >once a week/⩽once a week |
| —at age 25 | 0·57 (0·39–0·85) | 0·58 (0·35–0·98) | >once a week/⩽once a week |
| Cheese—now | 0·99 (0·68–1·45) | 1·08 (0·63–1·82) | >once a week/⩽once a week |
| —at age 25 | 0·69 (0·48–0·99) | 0·42 (0·25–0·70) | >once a week/⩽once a week |
| "Chal" at age 25† | 0·85 (0·49–1·45) | 1·25 (0·68–2·27) | ever/never |
| Sheep's yoghurt | 0·91 (0·56–1·45) | 1·03 (0·53–2·00) | any/0 |
| Green vegetables (cooked) | 0·81 (0·53–1·25) | 0·95 (0·53–1·69) | ⩾once a week/<once a week |
| Peas and beans (cooked) | 0·94 (0·66–1·33) | 0·68 (0·43–1·08) | >once a week/⩽once a week |
| Pumpkin (cooked) | 0·64 (0·43–0·95) | 0·67 (0·42–1·06) | ⩾once a week/<once a week |
| Cooked meals | 0·72 (0·50–1·04) | 0·87 (0·54–1·39) | ⩾once a day/<once a day |
| Raw green vegetables | 0·61 (0·42–0·87) | 0·57 (0·35–0·95) | ⩾once a week/<once a week |
| Raw garlic | 1·11 (0·77–1·59) | 0·80 (0·51–1·27) | ⩾once a month/<once a month |
| Raw tomatoes | 0·61 (0·43–0·86) | 1·08 (0·69–1·67) | >once a month/⩽once a week |
| Raw onions | 0·70 (0·49–1·01) | 0·79 (0·51–1·23) | >once a month/⩽once a week |
| Raw pickled garlic | 0·63 (0·44–0·89) | 1·06 (0·68–1·67) | ever/never |
| Wild vegetables | 0·85 (0·58–1·25) | 0·81 (0·49–1·33) | often in season/never or occasionally |
| Apples—now | 0·68 (0·47–0·98) | 0·50 (0·30–0·83) | >once a week/⩽once a week |
| —at age 25 | 0·63 (0·40–0·96) | 0·87 (0·52–1·45) | ever/never |
| Quince—now | 0·73 (0·49–1·09) | 0·65 (0·38–1·14) | ⩾once a week/<once a week |
| —at age 25 | 0·79 (0·52–1·20) | 0·85 (0·52–1.37) | ever/never |
| Grapes | 0·72 (0·50–1·04) | 0·68 (0·43–1·08) | >once a week/⩽once a week |
| Melons | 0·54 (0·32–0·91) | 0·67 (0·35–1·28) | most days/⩽once a week |
| Cucumbers | 0·51 (0·35–0·74) | 0·57 (0·35–0·94) | >once a week/⩽once a week |
| Plums—now | 0·54 (0·37–0·80) | 0·43 (0·24–0·75) | >once a week/⩽once a week |
| —at age 25 | 0·67 (0·44–1·01) | 0·63 (0·38–1·02) | ever/never |

(*contd.*)

TABLE IV. (*contd.*)

| Variable | Males n=217 | Females n=127 | Factor levels for which relative risk calculated |
|---|---|---|---|
| Consumption of: | | | |
| Cherries | 0·64 (0·41–1·00) | 0·62 (0·35–1·11) | ⩾once a week/<once a week |
| Oranges | 0·59 (0·41–0·84) | 0·46 (0·28–0·74) | >once a week/⩽once a week |
| Dried lemons | 0·52 (0·33–0·81) | 1·16 (0·63–2·13) | ever/never |
| Wild fruits | 0·54 (0·36–0·83) | 0·50 (0·24–1·00) | often/never or occasionally |
| Other fruits | 0·61 (0·38–0·95) | 0·56 (0·29–1·08) | ever/never |
| Drinking of hot tea | 1·59 (1·14–2·27) | 1·89 (1·22–2·94) | yes/no |
| Cigarette smoking | 1·52 (1·04–2·17) | 2·13 (0·68–6·67) | >5 a day/⩽5 a day |
| Waterpipe smoking | 1·25 (0·74–2·08) | 1·15 (0·43–2·94) | ever/never |
| Chewing of "nass"‡ | 0·87 (0·50–1·52) | — | ever/never |
| Consumption of "majoveh"§ | — | 0·47 (0·31–1·04) | ever/never |
| Height | 0·75 (0·46–1·20) | 0·61 (0·29–1·28) | males: >165/⩽165 cm<br>females: >160/⩽160 cm |
| Fuel used for cooking: | | | |
| kerosene | 1·47 (1·02–2·13) | 1·33 (0·84–2·13) | usually, sometimes/occasionally, never |
| gas | 0·59 (0·40–0·88) | 0·69 (0·43–1·11) | ,, ,, ,, |
| Use of dyes for wool | — | 1·59 (0·90–2·78) | ever/never |

* Sheep's tail fat.
† A drink made from a non-alcoholic fermentation of camels' milk.
‡ A mixture of tobacco and lime.
§ A special food taken during pregnancy by Turkoman women.

TABLE V.—*Socio-economic status of urban male patients and controls assessed from their occupation (or from the appearance of their house relative to others in the community where occupation information was unclassifiable or unavailable*.)*

| | Lower status from | | | Mid status from | | | Higher status from | | | Un-defin-able | Totals | |
|---|---|---|---|---|---|---|---|---|---|---|---|---|
| | occup.[1] | or house | total | occup.[2] | or house | total | occup.[3] | or house | total | status | occup. | total |
| Oesophageal cancer patients | 25 | 9 | 34 | 0 | 13 | 13 | 10 | 3 | 13 | 1 | 35 | 61 |
| Controls | 25 | 10 | 35 | 1 | 22 | 23 | 49 | 15 | 64 | 0 | 75 | 122 |
| Total | 50 | 19 | 69 | 1 | 35 | 36 | 59 | 18 | 77 | 1 | 110 | 183 |
| Other cancer patients | 5 | 6 | 11 | 1 | 7 | 8 | 13 | 1† | 14 | 0 | 19 | 33 |
| Controls | 11 | 7 | 18 | 1 | 9 | 10 | 26 | 13 | 39 | 1 | 38 | 66 |
| Total | 16 | 13 | 29 | 2 | 16 | 18 | 39 | 14 | 53 | 1 | 57 | 99 |

* The interviewers' assessment of the state of the house has been used as an index of socio-economic status for those with no urban occupation (farmers and farm workers) (16 oes. pat.; 37 controls; 5 other pat.; 14 controls); for those with unclassifiable occupations (drivers and salesmen and someone working in (? owning) a public bath) (5 oes. pat.; 4 controls; 4 other pat.; 5 controls); and for those with occupation unspecified (5 oes. pat.; 6 controls; 5 other pat.; 7 controls).

[1] Labourer; building labourer; shepherd; tinker; itinerant musician; street letter writer; servant; driver's assistant; fisherman; scrap-iron dealer; messenger; gardener; porter; cook; beggar.

[2] Plumber; painter.

[3] Teacher; government official; clerk; baker; butcher; blacksmith; shoemaker; tailor; carpenter; mechanic; shop owners or traders; bar owner; hotel owner; accountant.

$\chi^2_2$ (total urban residents) oesophageal cancer = 17·44 $P<0·001$.
other tumours = 2·42 N.S.

$\chi^2_1$ (urban residents with classifiable occupations, higher/lower) Oesophageal cancer 13·24 $P<0·001$.
Other tumours = 0·02 N.S.

Relative risk Higher socio-economic status/Lower socio-economic status: Oesophageal cancer 0·21, Other tumours 0·59 $P=0·09$**

Higher socio-economic occupations/Lower socio-economic occupations:
Oesophageal cancer 0·20, Other tumours 1·10 $P=0·03$**

** Exact test of the homogeneity of the relative risks.

† All the other cancer patients without classifiable occupations had cancer of the stomach, and the known higher risk of this tumour among persons of lower social class could explain the deficit of other tumour patients of higher socio-economic status judged from the state of their house.

economic status was asked of the village headman. The cases again appeared poorer than the controls.

*Ownership of land and animals*

Controls owned more cows than did cases, a difference that was more marked, among males, for ownership at age 25. There appeared to be an excess among the controls for ownership of sheep, but this was only marginally significant for men and not at all for women. For the other animals owned to any extent in the study area, goats, oxen, calves, camels and buffaloes, there was a slight but insignificant excess among controls.

Irrigated and unirrigated land were considered separately. There was little difference between cases and controls for the former; the latter showed a significant excess among the controls. In terms of land use, however, an even larger contrast lay in the growing of green vegetables which was, twice as frequent among controls than among cases.

No differences were seen in the frequency with which commercial crops were grown.

*Food consumption*

*Bread and rice.*—A range of questions were asked on the two staple foods of the region; no differences were seen between cases and controls.

*Meat, poultry and fish.*—No appreciable differences were seen between cases and controls.

*Fats.*—Consumption of butter, cheese and sesame-seed oil was higher among the controls, with a suggestion that differences in consumption at age 25 were more marked. The higher consumption of sesame-seed oil among controls is in interesting contrast to the geographical association of use of sesame oil with oesophageal cancer incidence, noted in the introduction.

*Other milk products.*—There were no appreciable differences in present consumption of milk or yoghurt, whether from cow, sheep or camel. There was an indication of higher consumption among controls of cows' milk at age 25, especially among males. The lack of association with consumption of sheep's milk or yoghurt contrasts with the geographical association, noted in the introduction.

*Vegetables.*—The majority of vegetables which are eaten cooked (potatoes, dried peas, beans, green vegetables, tomatoes, onions and egg plant) showed little difference between cases and controls. Vegetables eaten raw, salads, tomatoes, onions, garlic and pickled garlic, showed appreciable and significantly higher consumption among the controls. Wild vegetables showed little difference in consumption.

*Fruit.*—The majority of fruits, including wild berries, showed considerably greater consumption among the controls than among the cases.

*Fuel for cooking*

The main fuels used for cooking are kerosene and gas, available in cylinders. Wood of course is used throughout the area, but supplies are limited. Gas is used for preference and, as its introduction is recent, its use is an indication of higher present socio-economic status. Use of kerosene indicates a lack of means to avail oneself of a cleaner fuel. Gas is used more by controls, kerosene by the oesophageal cancer cases.

*Tea drinking*

The temperature at which tea is normally drunk appeared to be hotter among cases than controls (see, however, further analysis). The question asked was: "Normally, do you like to drink your tea very hot?" There was no difference in quantity usually drunk, as calculated from the replies on frequency and cup size, nor were there differences in the use of green tea, found only in the Turkoman area.

*Tobacco consumption*

The use of nass is confined to Turkoman males. There was no difference in consumption between cases and controls, nor was there an excess of water-pipe use among the cases. Both for males and females,

TABLE VI

| | Males Cigarettes/day | | | | Females Cigarettes/day | | | |
|---|---|---|---|---|---|---|---|---|
| | Never | 1–19 | 20+ | Total | Never | 1–19 | 20+ | Total |
| Oesaphageal ca. cases | 93 | 103 | 21 | 217 | 112 | 14 | 1 | 127 |
| Controls | 227 | 180 | 27 | 434 | 239 | 15 | 0 | 254 |
| Total | 320 | 283 | 48 | 651 | 351 | 29 | 1 | 381 |

$\chi^2$ for trend on 1 d.f.:
Males: $\chi^2 = 6{\cdot}16$, $P \simeq 0{\cdot}01$ Relative risk: heavy (20+) smokers/non-smokers = 1·90.
Females: $\chi^2 = 4{\cdot}07$, $P < 0{\cdot}05$ Relative risk: all smokers/non-smokers = 1·99.

there is a significant excess of cigarette smokers among the cases (Table VI). However, the groups mainly affected, that is heavy smokers among males and any smoker among females, amount to only 6% of the respective groups.

*Source of drinking water*

No significant differences between cases and controls.

*Home industries*

No local crafts were worked significantly more often by cases than controls, although there was a slight but non-significant excess of female cases who had ever used dyes.

*Anthropometric and clinical measurements*

An attempt was made to measure the height of individuals in the study, and questions were asked on previous symptoms of vitamin deficiencies, and on loss of teeth. Under the field conditions, the frequency of meaningful response was too low to warrant further analysis.

*Selection of variables for further analysis*

The clustering of "significant" results in Table IV among linked variables suggests that these were not chance correlations thrown up by the sheer number of comparisons made. For the remainder of the analysis, attention has been confined to representative variables from each grouping which appear to characterize most succinctly the differences between cases and controls.

These variables are:

Cigarette smoking
Drinking of hot tea
Proportion of children who have died
Number of years at school
Socio-economic status (village headman's assessment and/or state of the house)
Growing of green vegetables
Ownership of cows when aged 25
Number of living rooms
Use of kerosene for cooking
Use of gas for cooking
Present consumption of:
  raw green vegetables
  raw tomatoes
  oranges
  cucumbers
  dried lemon

*Limitation of analysis to cases with confirmed diagnosis*

21% of cases were diagnosed solely on clinical grounds. Analyses similar to those in the previous section were performed on the selected variables for the 79% of cases, and corresponding controls, for whom diagnosis was confirmed by either radiology or histology. The differences between the two analyses were small. In the analyses in succeeding sections, all cases have been included.

*Further analysis of the selected variables and comparison with other tumours*

In this section the analysis has taken full account of the matching, and the joint effect of groups of variables has been investigated, using regression methods as

TABLE VII.—*Relative risks for oeseophageal cancer, and for the other tumours in the study, associated with selected factors. Full account taken of the matching*

| Factor | Males: Relative risk for Oesophageal cancer n=217 | Males: Relative risk for Other tumours n=109 | Females: Relative risk for Oesophageal cancer n=127 | Females: Relative risk for Other tumours n=72 | $\chi^2$ on 1 d.f. for difference between relative risks[1] (oesophageal cancer/other tumours): Males | $\chi^2$ …: Females | Factor level for which relative risk is calculated |
|---|---|---|---|---|---|---|---|
| No of years at school | 0·40** | 0·56 | — | — | n.s. | — | any/0 |
| Proportion of dead children | — | — | 1·23* | 0·89 | — | 3·62 | >80%/60–79%/ 40–59%/20–39%/ <20% [2] |
| No. of living rooms | 0·56** | 0·85 | 0·52* | 0·69 | n.s. | n.s. | [3] |
| Socio-economic status | 0·23*** | 0·50* | 0·41** | 0·83 | n.s. | n.s. | very rich, rich, moderate/poor, very poor |
| Ownership of cows at age 25 | 0·41*** | 1·45 | 0·62* | 0·31** | 12·98 | 2·26 | any/0 |
| Growing of green vegetables | 0·39*** | 0·71 | 0·50* | 0·79 | 2·36 | n.s. | yes/no |
| Consumption of: | | | | | | | |
| raw green vegetables | 0·81*** | 0·85* | 0·85* | 0·86 | n.s. | n.s. | [4] |
| raw tomatoes | 0·83*** | 0·88* | 1·04 | 0·94 | n.s. | n.s. | [4] |
| cucumbers | 0·75*** | 0·85 | 0·79* | 0·85 | n.s. | 2·14 | [4] |
| oranges | 0·71*** | 0·76* | 0·69*** | 0·94 | n.s. | 3·50 | [4] |
| dried lemons | 0·68*** | 0·96 | 0·92 | 0·84 | 5·54 | n.s. | [4] |
| Drinking of hot tea | 1·72** | 3·23*** | 2·17** | 0·86 | 3·89 | 4·13 | yes/no |
| Cigarette smoking | 1·49* | 2·17** | 2·70 | 2·13 | n.s. | n.s. | >5/<5 |
| Fuel used for cooking: | | | | | | | |
| kerosene | 1·79** | 0·65 | 1·54 | 1·49 | n.s. | n.s. | sometimes, always/ occasionally, never |
| gas | 0·47*** | 0·65 | 0·63 | 0·49 | n.s. | n.s. | sometimes, always/ occasionally, never |

[1] Calculated from the difference in the log likelihood when combining both tumour types (see statistical analysis section).

[2] Coded as the integral part of (%died/20). (The risk changes by the given quantity for each unit step in the coded values. Thus relative risk for oesophageal cancer > 80% children died/<20% children died = $(1{\cdot}23)^4 = 2{\cdot}29$.

[3] Coded as 1 1 room

2 2 rooms

3 3 rooms

4 4 rooms or more

The risk changes as indicated in "2" above. Thus relative risk (male) for 4 or more rooms/1 room = $(0{\cdot}56)^3 = 0{\cdot}18$.

[4] Coded as 0 never
1 less than once a month
2 once a month
3 several times a month
4 several times a week
5 once a day
6 more than once a day

The risk changes as indicated in "2" above. Thus relative risk for every day/never (for raw green vegetables) = $0{\cdot}81^5 = 0{\cdot}35$).

* $0{\cdot}01<P<0{\cdot}05$ ** $0{\cdot}001<P<0{\cdot}01$ *** $P<0{\cdot}001$.

explained in the section on statistical analysis.

All other tumours have been grouped together to form a heterogeneous group of individuals in the Caspian Cancer Registry. The purpose is to investigate whether the findings for oesophageal cancer are specific for that tumour or represent rather the characteristics of those who seek treatment for, and are diagnosed with cancer (the types of cancer given in Table III).

In Table VII the relative risks for each of the selected variables are given for oesophageal tumours and all other tumours combined. Also displayed are the $\chi^2$ values with 1 d.f., comparing the two relative risks for each variable. The anomalous results for the drinking of hot

TABLE VIII.—*Regression coefficients* for 5 socio-economic variables when treated as a group oesophageal tumours, other tumours, and combined tumours, males*

| | Oesophageal cancer | other tumours | Oesophageal cancer and other tumours combined |
|---|---|---|---|
| Socio-economic status | −1·072 | −0·726 | −0·920 |
| Ownership of cows at age 25 | −0·553 | 0·573 | −0·216 |
| Growing of green vegetables | −0·859 | −0·239 | −0·640 |
| Use of kerosene for cooking | 0·544 | −0·508 | 0·200 |
| Use of gas for cooking | −0·259 | −0·531 | −0·356 |
| | Max. Log Lik. = −207·57 | Max. Log Lik. = −113·256 | Max. Log Lik. = −330·954 |

$\chi^2 = 20{\cdot}24$ $P \sim 0{\cdot}001$, for the difference between the two sets of regression coefficients ($= 2 \times (330.954 - 207{\cdot}57 - 113{\cdot}26)$).

* The regression coefficients are obtained by regressing the logarithm of the odds ratio on the independent variables, using the conditional (*i.e.* incorporating the matching) likelihood (Breslow *et al.*, 1978).

TABLE IX. *Association of oeophageal cancer with dietary factors after adjustment for socio-economic status**

(a) *Raw vegetables*

| | Regression coefficient | Relative risk (never/ several times a week) |
|---|---|---|
| Raw green vegetables | −0·0883 | 1·424 |
| Raw tomatoes | −0·1371 | 1·731 |

Max. log likelihood = 201·96

$\chi^2_2 = 11{\cdot}22$ $P < 0{\cdot}005$

(b) *Fruit*

| | Regression coefficient | Relative risk (never/ several times a week) |
|---|---|---|
| Oranges | −0·1552 | 1·860 |
| Cucumbers | −0·2223 | 2·433 |
| Dried lemon | −0·3192 | 3·585 |

Max. Log likelihood = 196·88
$\chi^2_3 = 23{\cdot}38$ $P < 0{\cdot}001$
* Using the same variables as in Table VIII.

tea reflect the association with gastric cancer, which in these data is stronger than the association with oesophageal cancer. Replies to questions on tea drinking may well be determined by the patient's developing gastrointestinal condition itself causing slight discomfort and awareness of the heat of foodstuffs, and no further attention will be paid in this article to the temperature at which tea is drunk. Otherwise, for males the relative risks for the other tumours are uniformly closer to unity than for oesophageal tumours. The lack of significance for many of the relative risks pertaining to the other tumours is partly due to the smaller sample size, but it is clear that the effects for at least some of the variables are significantly greater among the oesophageal cancer group. For females, with just over half the male sample size in each group, the effect is less apparent, but the trend is clearly the same. When the variables describing socio-economic status are treated as a group, the difference between oesophageal and other tumours is striking (see Table VIII with the results for males). For dietary items and for females, the differences are less clear, but again in the same direction. Table IX gives results for oesophageal cancer confined to males, showing that the effect of the 2 raw vegetables and of the 3 fruits, when taken as groups, remain significant even after adjusting for the effect of the 5 socio-economic variables. As before, the same trend is apparent for females, but the fewer numbers reduce the significance. The dietary variables were not significantly associated with the other tumours after adjusting for socio-economic status, but the lack of numbers renders the result inconclusive.

The conclusions one draws from the results of this section are:

(a) the increased risk associated with low socio-economic status is significantly greater for oesophageal tumours than for other tumours combined
(b) the effect of poor diet is significant for oesophageal tumours even after adjusting for socio-economic level

(c) the effects are seen more clearly when the matching is incorporated in the analysis.

*Demonstration of positive effects for lung cancer and breast cancer*

One purpose of the inclusion of other tumours in the study was to act as positive controls for the sensitivity of the questionnaire approach in the particular field setting. The strong association between lung, laryngeal and pharyngeal cancers and cigarette smoking is given in Table X.

TABLE X. *Cigarette-smoking association with lung, larynx and pharynx cancers (males)*

| | Number of cigarettes per day, present consumption | | | | |
|---|---|---|---|---|---|
| | 0 | 1–19 | 20–39 | 40 | Total |
| Lung cancer | 4 | 4 | 7 | 7 | 22 |
| Pharynx cancer | 0 | 2 | 1 | 1 | 4 |
| Larynx cancer | 1 | 0 | 3 | 0 | 4 |
| Total | 5 | 6 | 11 | 8 | 30 |
| Controls | 32 | 9 | 14 | 5 | 60 |
| Total | 37 | 15 | 25 | 13 | 90 |

Table XI gives parity and age at first birth for the breast-cancer cases and their controls. There is some association of risk with parity, but no appreciable effect for age at first birth. The latter may reflect the imprecision surrounding age in the region.

*Results of repeat interviewing*

Ninety-seven oesophageal cancer cases and the corresponding controls were revisited 12–18 months after the first interview, at which time a greatly shortened interview was used. Eighty-four cases (86·6%) had died, and on the majority of these occasions the spouse, if available, or other relative was asked to provide the information. Table XII compares the two sets of results. There are some discrepancies, but no indication of a systematic difference between cases and controls.

TABLE XII. *Comparison of the responses to the same question asked twice, 12 to 18 months apart*

| | Later response compared to earlier response | | |
|---|---|---|---|
| Question | Less | Same | More |
| Number of animals owned | | | |
| Case | 13 | 80 | 4 |
| Control | 24 | 151 | 14 |
| Consumption of raw green vegetables | | | |
| Case | 13 | 67 | 17 |
| Control | 42 | 117 | 30 |
| Size of garden | | | |
| Case | 13 | 64 | 20 |
| Control | 22 | 128 | 39 |
| Smoking history | | | |
| Case | 10 | 76 | 11 |
| Control | 12 | 168 | 9 |
| State of house | | | |
| Case | 22 | 51 | 24 |
| Control | 46 | 106 | 37 |

The number of controls is not precisely twice the number of cases. During the time available for reinterviewing 5 of the controls originally interviewed were not traced.

TABLE XI.—*Association of breast cancer with (a) parity, and (b) age at first birth*

(a) Parity

| | <4 | 4–6 | 7–10 | 11+ | Total |
|---|---|---|---|---|---|
| Breast cancer | 7 | 8 | 9 | 1 | 25 |
| Control | 7 | 16 | 21 | 6 | 50 |
| Total | 14 | 24 | 30 | 7 | 75 |

(b) Age at first birth

| | <18 | 18–20 | 21–24 | 25+ | Nulliparous | Unknown | Total |
|---|---|---|---|---|---|---|---|
| Breast cancer | 8 | 4 | 7 | 3 | 3 | 0 | 25 |
| Control | 13 | 17 | 16 | 3 | 0 | 1 | 50 |
| Total | 21 | 21 | 23 | 6 | 3 | 1 | 75 |

### DISCUSSION

Before discussing the substance of the results presented in the previous sections, the degree of reliability of the observations requires comment. The Caspian Cancer Registry has to contend with a thoroughly decentralized system of health care, there being no major treatment or diagnostic centres in the study area. Under these circumstances, only the existence of a well-functioning cancer registry made it possible to find the patients. Even so, a considerable number of the addresses given were inadequate. The diagnostic facilities in the region are not those of a developed economy, and the oesophagus is one of the very few sites at which the diagnosis of tumours is adequate. Further, the population naturally mistrusts those who arrive at their home in some official capacity to ask a long series of questions. In the replies, evasion, prevarication and lying will all occur to some extent and, under these circumstances, there is danger of bias and unreliable responses.

We would put forward the following reasons for supposing that the major elements of our findings are not artefacts. First, radiology is an acceptable form of diagnosis for cancer of the oesophagus, and together with histology formed the basis of diagnosis for almost 80% of cases. The results for all cases were similar to those for these 80%, so errors in diagnosis are not likely to have vitiated the results. Second, the possible biases arising from the selection of cases would probably have reduced rather than augmented the observed socio-economic association. As shown in Table I, the major cause of omitting registered cases which could have introduced bias is inadequate addresses, and it is the poorer patients for whom address information is most likely to be missing. The higher proportion with inadequate addresses among the other tumour patients could have biased the comparisons between the oesophageal cancer patients and this group of controls. However, it is suggested that differences in the origin of the records for the different tumours reduce the possibilities of bias. The majority of patients with cancer of the oesophagus are reported by the few centres with radiology facilities. These have a high standard of cooperation in the survey and it is likely that only those patients who themselves have difficulty in specifying their address will be reported without adequate details. By contrast, patients with the other tumours are reported more by the numerous general practitioners and doctors from small hospitals throughout the area who see perhaps 2 or 3 cases of cancer a year and may take less trouble in completing the unfamiliar cancer form. For these patients careless reporting is more likely to account for inadequate addresses.

Furthermore, any biases which arose from the interview setting, a village or small town of rural Iran, would have affected oesophageal cancer cases and other tumours equally. However, the results show unequivocally that the risk associated with low socio-economic status is increased specifically for the oesophagus. The results for poor dietary intake are not so clearcut, but point in the same direction. The fact that many of the other tumours are of the stomach makes this result even more striking. Lastly, the reliability of answers to the questionnaire is demonstrated at least to some extent by the positive findings both for cigarette smoking with cancers of the lung, pharynx, larynx and oesophagus, and for parity with breast cancer, and by the degree of concordance obtained on repeat interviewing.

The findings of the study fall into two groups, positive and negative. The importance of the latter lies in the possibility of eliminating a number of factors previously suspected of playing some role in the development of the disease, thereby narrowing the range of factors for further consideration. Of the variables listed in the introduction, the present results clearly show a lack of association with disease at the individual level for the following variables:

—consumption of sheep's milk and yoghurt: negative finding supported by the slightly greater ownership of sheep among the controls;
—use of sesame oil for cooking
—the chewing of nass: there was a positive association with cigarette smoking, but the negligible role of this factor in the epidemiology of the disease is shown by the attributable risk, $\sim 5\%$;
—making of carpets or felts;
—consumption of special foodstuffs during pregnancy: this finding is corroborated by the slightly lower parity of the cases;
—preserving of meat by salting and sun-drying;
—the use of wild spinach: cases used wild vegetables less than controls.

We are left, then, with consumption of bread, a low intake of various foods, use of opium and various aspects of tea consumption.

The main positive finding is the relatively low socio-economic status of the cases, associated with a low intake of a range of fruits and raw vegetables. Studies in other areas of the world have shown that oesophageal cancer cases are often poorer than the general population (Wynder & Bross, 1961; Martinez, 1969; de Jong *et al.*, 1974). However, the complexity of the societies in which these studies were performed obscures any clear interpretation of the role socio-economic differences might play. The variety of cultural subgroups, and the relationship of both socio-economic status and frequency of a variety of exposures to these subgroupings, gives rise to potentially important confounding. The interest in the present result lies in the difference emerging even when the controls were matched for village of residence and language group. The heterogeneity within a village is not great, and socio-economic stratification is related to only a small range of variables.

A feature of the socio-economic association is that objective measures of earlier economic status show as strong an association as measures of present status. The proportion of children who died refers in most cases to a period 20 or more years before the diagnosis of the disease. Attendance at school is even earlier in an individual's life. Several other measures, such as ownership of animals or consumption of various foods, indicated a stronger association at age 25 than at the present. These latter measures on their own would need cautious interpretation, but taken together with the more objective measures would indicate a long-lasting poverty. Thus the low socio-economic status found among the cases is certainly not a post-disease phenomenon (*i.e.* not a reflection of the impoverishment serious disease can entail in such a society).

One factor clearly related to low socio-economic status is diet, and in particular, as shown in this study, low consumption of fresh vegetables and fruit. The strong negative association with growing green vegetables corroborates this association.

The results of the earlier study (Joint Iran/IARC Study Group, 1977) showed that the high-incidence area suffered a severe lack of both fruit and vegetables, and also that the Turkoman population has no interest in raw vegetables and salads, even when readily available. The results suggest to us that dietary inadequacy of this kind increases risk of oesophageal cancer.

A second factor potentially associated with socio-economic class within a village is the continued use of traditional but outmoded agricultural practices. In particular, the storage of wheat in underground pits (see Joint Iran/IARC Study Group, 1977) and inefficient methods of separating foreign seeds from the wheat. Both factors could lead to higher contamination of the bread of the poorer strata of the population. The results of the case-control study show no differences in quantity of bread consumed, but there is no direct measure of degree of contamination from this study. However, considerable work has now been done on identifying the contaminants of the local wheat, whether seeds or fungi, and the results indicate little possibility of contamination with potential carcinogens (IARC, 1978).

Opium addiction is often associated with low socio-economic status, the addiction itself absorbing a large proportion of the addict's resources. The results of this study are thus consistent with a role for opium addiction. In this respect, the mutagenic activity of both crude opium and opium residues as shown by the Ames test is of considerable significance (Hewer *et al.*, 1978, submitted for publication).

The tentative conclusion from the results of a previous study (Joint Iran/IARC Study Group, 1977) was that the very high risk for oesophageal cancer in north-east Iran arises from a severely limited diet, in conjunction with exposure to a carcinogenic agent probably present in opium or in wheat contaminants. The present study provides evidence on an individual basis to support this conclusion. In conjunction with the results of other studies, on wheat contaminants and on opium, the hypothesis supported by most aspects of the data is that the high risk is mediated by exposure to components of opium in conjunction with a diet very low in fruit and fresh vegetables.

We are grateful to Dr J. Kmet, Dr C. S. Muir and Dr A. Nadim for continual encouragement and support. We express our thanks to Messrs Alaghi, Ebadi, Eftehari, Gholami, Golchubian, Jonedi, Kur, Lotfalipur, Mozaffari, Sadeghpur, Satari, Safarnejat, Sardari, Shahhosseini and Tala'i for their work in the field; to Messrs Ahmadi, Peighambari and Jalapur for cooperation from the Babol Cancer Registry; to Messrs Saeb, Tadjbakhsh and Amini for their work in processing the field data and to Mrs G. Dahanne and Mrs J. Rezaie for the typing of several drafts of the paper. The project was supported by Public Health Service Contracts NO1-CP43342 and NO1-CP71048 from the Division of Cancer Cause and Prevention, National Cancer Institute.

### REFERENCES

BRESLOW, N. E., DAY, N. E., SABAI, S. and HALVORSEN, K. T. (1978) Estimation of multiple relative risk functions in marked case-control studies. *Am. J. Epidemiol.*, **108**, 299.

COX, D. R. Regression models and life tables (with discussion) (1972) *J. R. Statist. Soc.*, **34**, 187.

DE JONG, U. W., BRESLOW, N., GOH EWE HONG, J., SRIDHARAN, M. & SHANMUGARATNAM, K. (1974) Aetiological factors in oesophageal cancer in Singapore Chinese. *Int. J. Cancer*, **13**, 291.

GART, J. J. (1971) The comparison of proportions: a review of significance tests, confidence intervals and adjustments for stratification. *Int. Stat. Inst.* **39**,

HEWER, T. F., ROSE, E., GHADIRIAN, P., BARTSCH, H., MALAVEILLE, C. & DAY, N. (1978) Ingested mutagens from opium and tobacco and cancer of the oesophagus. *Lancet*, ii, 494.

IARC (1978) *Annual Report 1977*. Lyon: World Health Organization.

JOINT IRAN/IARC STUDY GROUP (1977) Esophageal cancer studies in the Caspian littoral of Iran: results of population studies—a prodrome. *J. Natl Cancer Inst.*, **59**, 1127.

KMET, J. & MAHBOUBI, E. (1972) Oesophageal cancer in the Caspian littoral of Iran: initial studies. *Science*, **175**, 846.

MAHBOUBI, E., KMET, J., COOK, P. J., DAY, N. E., GHADIRIAN, P. & SALMASIZADEH, S. (1973) Oesophageal cancer studies in the Caspian littoral of Iran: the Caspian Cancer Registry. *Br. J. Cancer*, **28**, 197.

MANTEL, N., BROWN, C. & BYAR, D. P. (1977) Tests for homogeneity of effect in an epidemiologic investigation. *Am. J. Epidemiol.*, **106**, 125.

MANTEL, N. & HAENSZEL, W. (1959) Statistical aspects of the analysis of data from retrospective studies of disease. *J. Natl Cancer Inst.*, **22**, 719.

MARTINEZ, I. (1969) Factors associated with cancer of the esophagus, mouth, and pharynx in Puerto Rico. *J. Natl Cancer Inst.*, **42**, 1069.

WYNDER, E. L. & BROSS, I. J. (1961) A study of etiological factors in cancer of the esophagus. *Cancer*, **14**, 389.